# Divergent targets of glycolysis and oxidative phosphorylation result in additive effects of metformin and starvation in colon and breast cancer

**DOI:** 10.1038/srep19569

**Published:** 2016-01-22

**Authors:** Cecilia Marini, Giovanna Bianchi, Ambra Buschiazzo, Silvia Ravera, Roberto Martella, Gianluca Bottoni, Andrea Petretto, Laura Emionite, Elena Monteverde, Selene Capitanio, Elvira Inglese, Marina Fabbi, Francesca Bongioanni, Lucia Garaboldi, Paolo Bruzzi, Anna Maria Orengo, Lizzia Raffaghello, Gianmario Sambuceti

**Affiliations:** 1 CNR Institute of Molecular Bioimaging and Physiology (IBFM), Milan, Section of Genoa, Genoa, Italy; 2 Oncology lab, Istituto Giannina Gaslini, Genoa, Italy; 3 Nuclear Medicine, Department of Health Sciences, University of Genoa and IRCCS AOU San Martino-IST, Genoa, Italy; 4 Biochemistry Lab, Department of Pharmacy, University of Genoa, Genoa, Italy; 5 Core facility, Istituto Giannina Gaslini, Genoa, Italy; 6 Animal facility, IRCCS AOU San Martino-IST, Genoa, Italy; 7 Integrated oncological therapies, IRCCS AOU San Martino-IST, Genoa, Italy; 8 Statistics and Epidemiology Unit, IRCCS AOU San Martino-IST, Genoa, Italy

## Abstract

Emerging evidence demonstrates that targeting energy metabolism is a promising strategy to fight cancer. Here we show that combining metformin and short-term starvation markedly impairs metabolism and growth of colon and breast cancer. The impairment in glycolytic flux caused by starvation is enhanced by metformin through its interference with hexokinase II activity, as documented by measurement of 18F-fluorodeoxyglycose uptake. Oxidative phosphorylation is additively compromised by combined treatment: metformin virtually abolishes Complex I function; starvation determines an uncoupled status of OXPHOS and amplifies the activity of respiratory Complexes II and IV thus combining a massive ATP depletion with a significant increase in reactive oxygen species. More importantly, the combined treatment profoundly impairs cancer glucose metabolism and virtually abolishes lesion growth in experimental models of breast and colon carcinoma. Our results strongly suggest that energy metabolism is a promising target to reduce cancer progression.

Cancer energy metabolism is regulated to support high-energy demand and increased biosynthesis of macromolecules for a rapidly growing biomass. As in most tissues with high proliferative activity, this metabolic pattern commonly implies a high glycolytic flux facing relatively low rates of oxidative phosphorylation (OXPHOS) regardless an adequate tissue oxygen tension. This phenomenon known as “Warburg effect” is particularly functional for cancer eventually slowing OXPHOS whose generation of reactive oxygen species (ROS) would inevitably hamper cellular redox status and thus DNA replication^1^.

Due to these features, this metabolic reprogramming has to face the relatively low efficient ATP production allowed by glucose conversion to lactate. This limitation is most often overcome by an enhanced expression of glucose transporters increasing glycolytic flux as to enable cancer to rapidly deplete glucose from the surrounding tissues^2^. Nevertheless, this task is often difficult to achieve in the hypo-vascular microenvironment of a rapidly growing mass and contributes to the frequent occurrence of necrotic core in large cancer lesions^3^.

According to this consideration, targeting energy metabolism represents a promising tool in anti-cancer therapy. In this line, short-term starvation (STS) has been found to profoundly impair cancer growth at least partially by down-regulating glycolytic flux and increasing cell dependency on OXPHOS^4^. This metabolic reprogramming inevitably increases oxygen consumption rate (OCR) and ROS production^5^,^6^ whose toxic consequences are more pronounced for proliferating cancer cells with respect to normal tissues^7^. However, these effects are intrinsically transient and more severe impairments in energy metabolism are required to prevent growth rebound after re-feeding^8^.

Recent evidence indicates that metformin (MTF) – the most widely used drug in treatment of type 2 diabetes – might represent a suitable tool to this purpose. On one side, drug-induced reduction in hepatic gluconeogenesis and glucose release in the bloodstream^9^,^10^,^11^ should limit liver buffer function and amplify the decrease in serum glucose level during periods of reduced nutrients intake. On the other hand, MTF has been shown to hamper cancer fuelling via a direct inhibition of mitochondrial respiratory Complex I that might complement energy depletion caused by STS^12^,^13^. In agreement with this hypothesis, several experimental studies documented a significant reduction in ATP synthesis in MTF-treated cancer models resulting in reduced cell proliferation up to cell death^14^,^15^,^16^. Vice versa, the low glucose availability caused by STS would prevent the increase in glycolytic flux described in cancer cells in response to OXPHOS inhibition by MTF through its interference with Complex I function^12^,^17^.

The present study was designed to verify the potential of combining MTF and STS in reducing cancer growth in murine models of colon (CT26) and breast (4T1) carcinoma. This hypothesis was first verified *in vivo* by imaging studies documenting the systemic reaction to the metabolic perturbation and the additive interference of MTF and STS on metabolism and growth of both cancer models. Thereafter, the underlying mechanisms were evaluated in cell cultures in which the combined treatment coupled a profound and simultaneous alteration in glycolytic flux and OXPHOS, eventually combining oxidative damage and ATP depletion as to impair cell viability and proliferation.

## Results

### Metformin and starvation affect cancer growth and glucose metabolism *in vivo* models

*In vivo* experiments confirmed the hypothesis that MTF, STS and their combination remarkably reduce cancer growth. As a first step, toxic profile of STS + MTF was verified in twelve animals submitted to the inoculation of 200 000 CT26 cells and followed for 15 days without any other manoeuvre with (n = 6) or without (n = 6) combined treatment. For imaging study, each cancer model was subcutaneously implanted in four mice groups (control n = 6, STS n = 6, MTF n = 6, and merged treatment n = 6) accounting for a total of 48 studied animals. The studies were completed in all animals and no side effects occurred at the drug dosage used. Body weight was not modified by MTF while it showed a transient decrease at the end of STS regardless drug treatment. On the contrary, serum glucose concentration was not affected by MTF, it was slightly and not significantly reduced by STS and reached its lowest values in mice exposed to the combined treatment before both imaging studies (Supplementary Table 1).

**Figure 1:**
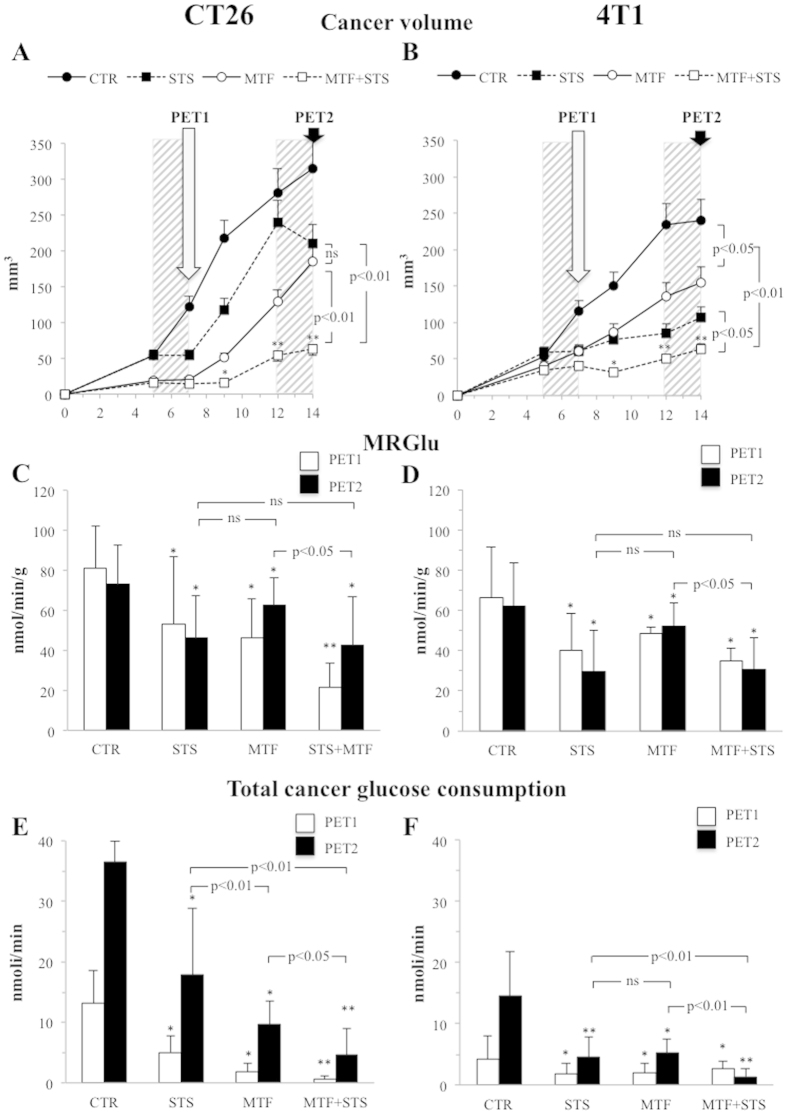
MTF and STS effect on cancer growth and glucose metabolism. (**A,B**): cancer progression throughout the whole study period (14 days), respectively in CT26 and 4T1 mice. Average tumour volumes are expressed in cubic millimetres. White and black arrows correspond to PET1 and PET2 scans while dashed grey columns indicate STS. The effect of merged treatment on tumour growth was more powerful than each intervention alone in both cancer models leading to a profound reduction in cancer progression whose significance was reached at day 9 and further increased until day 14 with respect to controls. (**C,D**) represent MRGlu evaluation at PET1 (white columns) and PET2 (black columns). This parameter was significantly reduced by all treatments without significant difference between each intervention. The simultaneous evaluation of cancer volume and MRGlu through the total cancer glucose consumption analysis demonstrated an additive effect of MTF and STS on both CT26 (**E**) and 4T1 (**F**). Data are presented as mean ± SD. *p < 0.05; **p < 0.01 (statistical differences vs controls). Black lines: statistical differences between each stressor at PET2).

Both tumour models became visible and palpable at day #5 after subcutaneous implantation in control animals. At subsequent monitoring, growth rate was higher for CT26 (Fig. 1A) with respect to 4T1 (Fig. 1B) lesions while it was comparably reduced by MTF. Similarly, first STS application stopped cancer expansion that, however, reaccelerated in the re-feeding period in both models. This rebound phase was particularly evident in CT26 animals (Fig. 1A) while it was largely smoothed in 4T1 ones (Fig. 1B). In both experiments, repeated STS again interrupted lesion growth confirming the transient nature of STS effect.

Despite these differential responses, combining MTF and STS virtually flattened tumour growth throughout the study in both cancer types. In fact, volume reduction with respect to control animals reached the statistical significance already at day #9 and became progressively more evident up to experiment termination at day #14, both in CT26 mice (63 ± 50 mm^3^ *vs* 315 ± 180 mm^3^, respectively, p < 0.001) and in 4T1 ones (63 ± 20 mm^3^ *vs* 240 ± 80 mm^3^, respectively, p < 0.001) (Fig. 1A,B). No significant interaction between the two treatments could be documented (Supplementary Figure 1A). Treatment effect on tumour growth (Supplementary Figure 1B) was confirmed by the analysis of the six animals not submitted to radionuclide imaging after CT26 inoculum.

Dynamic microPET scanning with ^18^F-fluoro-deoxyglucose (FDG) documented that tumour metabolic response preceded the growth reduction: at day #7, both MTF and STS significantly reduced lesion glucose consumption (MRGlu) in both CT26 (Fig. 1C) and 4T1 (Fig. 1D) models. This response persisted for the whole experiment particularly in the presence of STS. At both imaging times (day #7 and #14), synergism analysis reported a negative interaction between the two interventions, suggesting that both MTF and STS actually acted on the same metabolic pathways (Supplementary Figure 1C-D). A similar consideration also applied to total cancer glucose consumption that mostly decreased in mice exposed to the combined treatment in both cancer models (Fig. 1E,F) again with a negative interaction between the two treatments (Supplementary Figure1 E-F).

**Figure 2:**
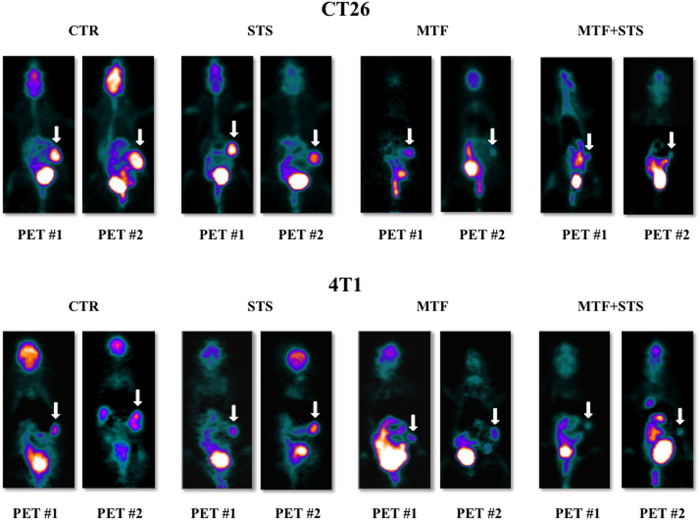
Parametric maps of glucose consumption (MRGlu). Here is appreciable, at a glance, the effect of the different treatments on CT26 (upper panels) and 4T1 (bottom panels) cancer models. Both STS and MTF affected cancer metabolism and growth. The most evident effect was obtained under combined treatment, in which tumor progression between the two scans was virtually abolished.

Intriguingly, the large disparity in tumour metabolism observed at parametric maps of glucose consumption (Fig. 2), was markedly less evident in conventional images in which the high variability of tracer retention smoothed the differences in standardized values of tracer uptake (SUV) among the tested protocols (Supplementary Figure 1G).

### Direct effect of metformihn and starvation on cancer glucose consumption

**Figure 3:**
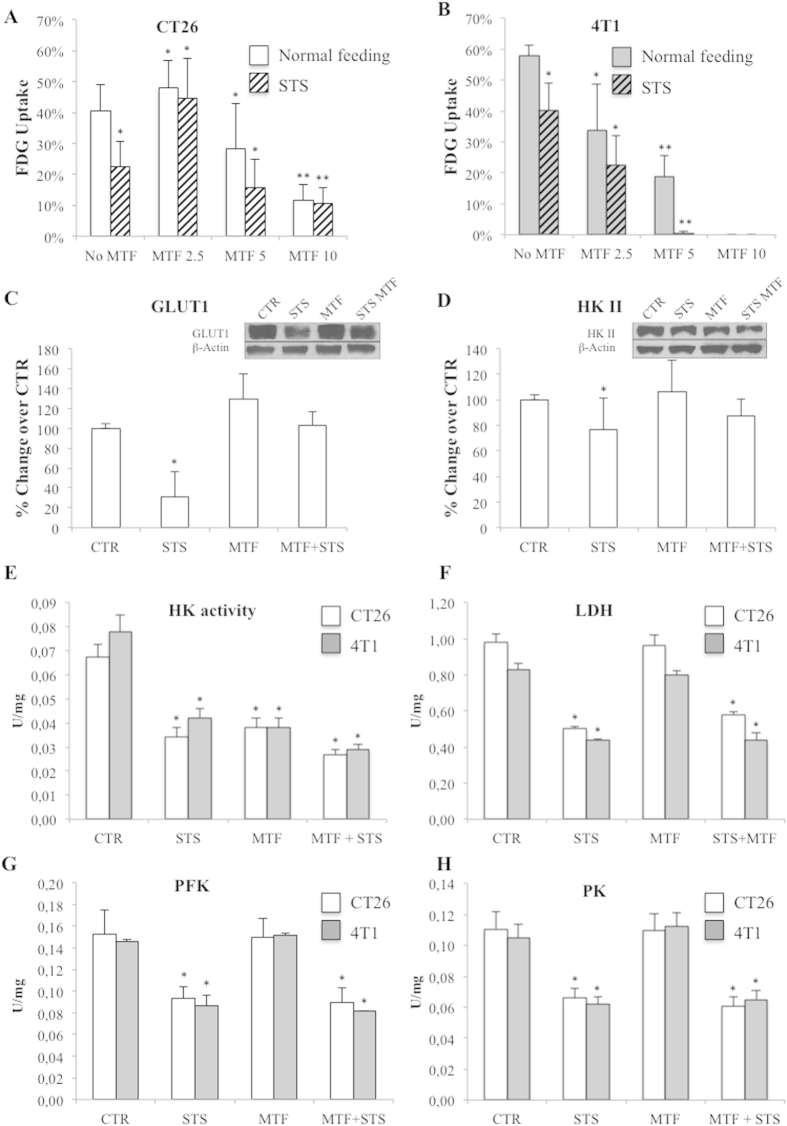
Effects of MTF and STS on the main regulators of glucose consumption. (**A**) After a modest increase at the lowest MTF dose (2.5 mM), FDG uptake in CT26 (white columns) progressively decreased down to its minimum value at 10 mM concentration. (**B**) In 4T1 cells (gray columns), tracer retention was already reduced at the lowest concentration (2.5 mM), while the effect of 10 mM dose could not be documented because cells viability was completely lost. In both CT26 and 4T1 cells the drug effect was empowered by STS (dashed columns) that reduced FDG uptake in all experimental conditions. (**C,D**) Western blot evaluation in CT26 cells demonstrated that GLUT1 and HK II expression were reduced only in presence of STS. (**E**) On the contrary, HK II function was significantly and similarly reduced by all tested protocols in both cell lines, reaching its lowest value under merged treatment. (**F**) LDH activity showed an almost exclusive response to STS. (**G,H**) STS induced a significant reduction of PFK and PK enzymatic activities in both cell lines, independently from MTF. Data are presented as mean ± SD; (*p < 0.05; **p < 0.01 statistical differences vs controls).

To rule out the confounding effect of systemic feedback mechanisms, we evaluated MTF and STS effect on glucose consumption by measuring FDG uptake in cultured cells using the same factorial experimental design of the *in vivo* study. In cancer cells, MTF decreased glucose consumption in a dose dependent fashion. Actually, this effect was slightly different in the two cell lines with CT26 cells less sensitive with respect to 4T1 cells (Fig. 3A,B) in which response to 10 mM could not be documented for a complete loss of cell viability. Intriguingly, this drug action was enhanced by STS that further reduced FDG uptake under all experimental conditions in both cell lines (Fig. 3A,B) while synergism analysis confirmed that the two interventions acted on similar targets as suggested by the negative sign of their interaction mostly evident in CT26 line (Supplementary Figure 3A-B). By contrast, metabolic response of non-cancer cells (human fibroblasts) was completely different since both MTF (5 mM) and STS caused a significant increase in FDG uptake (Supplementary Figure 2A).

To explain the mechanisms underlying the evident reduction in glucose avidity caused by both treatments in cancer cells, we first evaluated the availability of proteins related to glycolysis using a proteomic approach. The analysis was performed by Label Free Quantitation (LFQ) High Resolution/Mass Accuracy Liquid Chromatography Tandem Mass Spectrometry (HR/MA LC MS/MS), as described in detail in Supplementary Methods. Supplementary Figure 4A shows a representative heat map of selected Gene Ontology (GO) biological processes related to glycolysis. Interestingly, the two stressors induced a similar impairment in glucose metabolism despite an opposite response of glycolytic enzymes asset that was depleted by STS and empowered by MTF.

Accordingly, we directly tested with Western blot analysis the key determinants of glycolytic flux in cancer. Expression of both GLUT1 (Fig. 3C) and HK II (Fig. 3D) did not explain FDG uptake response, since availability of these proteins was reduced only by STS through a mechanism largely prevented by MTF (Supplementary Figure 3C-D). However, total cell lysate HK activity was similarly reduced in both cell lines by all tested protocols (Fig. 3E and Supplementary Figure 3E). Moreover, a completely different response was observed for lactate dehydrogenase (LDH, Fig. 3F) and for the rate-limiting steps of glycolysis catalysed by phosphofructokinase (PFK) and pyruvate kinase (PK) (Fig. 3G,H): the catalytic function of these enzymes was selectively impaired by STS being virtually not responsive to MTF (Supplementary Figure 3F-H) in both cancer models. Differently from MTF, whose action was similar in neoplastic and normal cells, STS effect on HK, PK and LDH activity was only trivial in fibroblasts (Supplementary Figure 2B-D).

**Figure 4:**
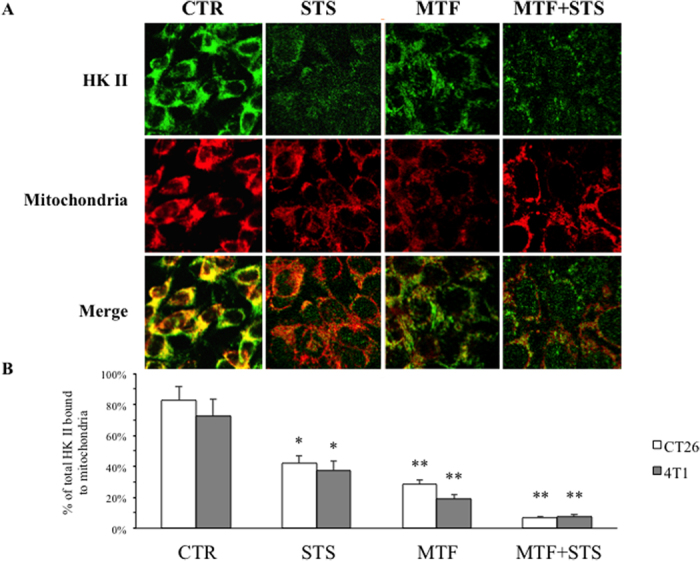
Immunofluorescence analysis in CT26 cells. (**A**) Confocal microscopy for HK II and mitochondria in CT26 cells under control condition, STS, MTF 5 mM for 24 hours and under MTF + STS. Mitochondria were labeled by MitoTracker Far Red; HKII was stained by indirect immunofluorescence, using a FITC-conjugated secondary antibody. First, second and third rows show staining for HK II, mitochondria and both, respectively. Merge images (third row) document that STS and MTF caused a significant and selective dislocation of HK II from mitochondrial membrane to the cytosol, mostly evident under combined treatment. (**B**) Percentage of total HK II bound to mitochondria showed a progressive reduction under STS, MTF and combined treatment in both CT26 (white columns) and 4T1 cells (gray columns). Data are presented as mean ± SD; (*p < 0.05; **p < 0.01 statistical differences vs controls).

Accordingly, the effect and the relative interactions of MTF and STS on cancer glycolytic rate were explained by the fact that the two interventions interfered with different targets of the same glycolytic pathway: MTF directly hampered HK II function while STS selectively impaired PFK and PK activity. The consequent reduction in glucose consumption was further enhanced by a relative de-localization of HK II away from the outer mitochondrial membrane that was documented by immunofluorescence analysis (Fig. 4). This response and the consequent loss of preferential access to ATP for glucose phosphorylation^18^ actually occurred under the two treatments and mostly under their combination.

### Effect of metformin and starvation on cancer energy metabolism

**Figure 5:**
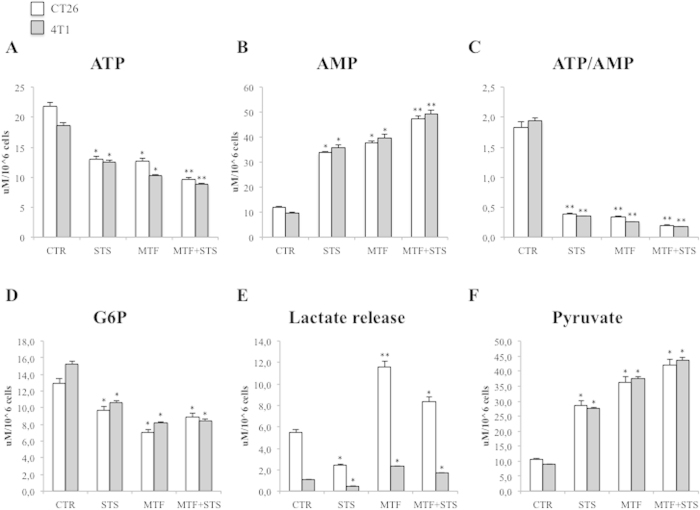
Cancer cell energy asset and glycolytic pathways. (**A**) ATP content in CT26 (white columns) and 4T1 (grey columns) cells was significantly reduced by STS, MTF and mostly by their combination. This effect was coupled by an increase in AMP concentration (**B**) and, thus, by an evident reduction in ATP/AMP ratio (**C**). The metabolic pathways of these findings are depicted below: (**D**) shows a significant reduction in G6P levels, mostly evident under MTF. This effect was paralleled by a significant increase in lactate release induced by the biguanide with an opposite response to STS (**E**). This different action was not explained by pyruvate availability whose concentration was increased by all interventions (**F**). Data are presented as mean ± SD; (* p < 0.05; ** p < 0.01 statistical differences vs controls).

The impairment in glycolytic flux induced by MTF and STS was paralleled by a significant reduction in intracellular ATP concentration (Fig. 5A), by a marked increase in AMP (Fig. 5B) and thus by an obvious reduction in ATP/AMP ratio (Fig. 5C) in both models of colon and breast carcinoma. Again, combining the two treatments caused the most severe energy depletion, though with a negative interaction (Supplementary Figure 5A-C). This effect was only partially reproduced in fibroblasts (Supplementary Figure 2E).

The role of glycolysis as a common target of both interventions was confirmed by the evident response of glycolytic intermediates and end product. As shown in Fig. 5D, cytosol concentration of glucose-6-phosphate (G6P) moderately decreased under STS and fell down to its lowest values in response to MTF, confirming the role of HK II inhibition caused by the biguanide. On the contrary, lactate release in culture medium had a completely dissimilar behaviour: it was markedly increased by MTF and significantly reduced by STS (Fig. 5E) without any interaction between the two treatments (Supplementary Figure 5E).

This divergent response occurred despite a preserved availability of pyruvate whose cytosol concentration was increased by all interventions up to its maximal value under merged treatment (Fig. 5F, Supplementary Figure 5F). These data thus indicated that although both stressors actually acted on glycolytic rate, they determined a different fate of intracellular glucose within cancer cell: STS preserved its conversion to acetyl-CoA for Krebs cycle utilization, while MTF markedly increased its conversion to lactate. In other words, these experiments suggested that cell respiration was enhanced by STS and impaired by MTF.

**Figure 6:**
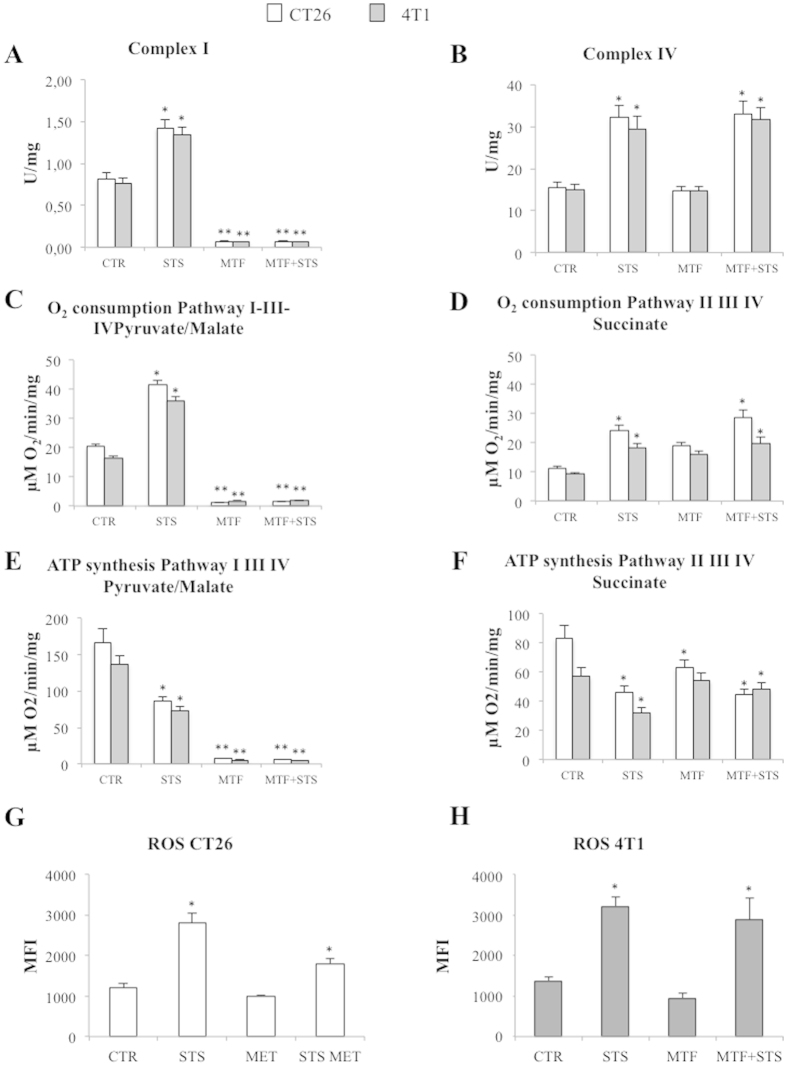
Effects of MTF and STS on OXPHOS and ROS production. (**A**) profound inhibition exerted by 5 mM MTF and merged treatment on mitochondrial complex I activity in both CT26 (white columns) and 4T1 (grey columns) cells. By contrast, STS led to an increase in Complex I and IV activity, independently from MTF (**A–C**). OCR response to pyruvate/malate administration (interrogating I-III-IV pathway) was virtually abolished after exposure to MTF and merged treatment while it was increased by STS alone. (**D**) STS slightly increased OCR response pathway II-III-IV activity as evaluated by succinate administration, independently from the presence of MTF. (**E**) ATP synthesis in the presence of pyruvate/malate was reduced by STS and virtually abolished by MTF and MTF + STS. (**F**) In the presence of succinate, ATP synthesis was slightly though significantly reduced by MTF and merged treatment. ROS production was enhanced by STS regardless MTF administration in both CT26 (**G**) and 4T1 (**H**) explaining the mismatch between increased OCR and reduced ATP synthesis. Data are presented as mean ± SD; (*p < 0.05; **p < 0.01 statistical differences vs controls).

To elucidate the mechanisms underlying the divergent fate of G6P we repeated a proteomic evaluation of major OXPHOS components (Supplementary Figure 4B). At this analysis, protein abundance of respiratory chain elements was reduced by STS and was increased by MTF despite the marked increase in lactate release induced by the biguanide. Evaluation of OXPHOS activity explained this apparent paradox. Actually, Complex I activity was virtually abolished by 5 mM MTF that also prevented the boost induced by STS (Fig. 6A). On the contrary, Complex IV was enhanced by STS while it was not influenced at all by MTF (Fig. 6B). Although Complex II function did not respond to either treatment, these divergent actions resulted in largely different functional consequences (Supplementary Figure 6A-B). MTF virtually abolished oxygen consumption rate (OCR) blocking pathway I-III-IV (Fig. 6C and Supplementary Figure 6C). By contrast, STS markedly increased OCR enhancing both respiratory pathways with its effect persisting under merged treatment only on pathway II-III-IV (Fig. 6D and Supplementary Figure 6D). Again, STS appeared to selectively affect cancer cells since Complex IV function remained unchanged under all treatments in fibroblasts, while MTF action on Complex I was reproducible in all studied cells (Supplementary Figure 2F-G).

The consequences of this functional impairment were even more evident when energy balance was analysed. ATP synthesis through pathway I-III-IV was virtually abolished by MTF independently from STS (Fig. 6E, Supplementary Figure 6E). On the other hand, its production through pathway II-III-IV was significantly impaired by STS despite the increased OCR (Fig. 6F, Supplementary Figure 6F). The mechanisms underlying this mismatch was elucidated by the measurement of reactive oxygen species (ROS), whose production was enhanced by STS independently from MTF administration in both CT26 (Fig. 6G) and 4T1 (Fig. 6H) cell lines (Supplementary Figure 6G).

Accordingly, STS and MTF caused a severe depletion of cancer cell energy asset through different actions on mitochondrial respiratory chain: the former mostly uncoupled OXPHOS and ATP synthesis, the latter markedly and selectively impaired cell respiration through a severe inhibition of Complex I.

### Cancer biological response to energy depletion caused by metformin and starvation

**Figure 7:**
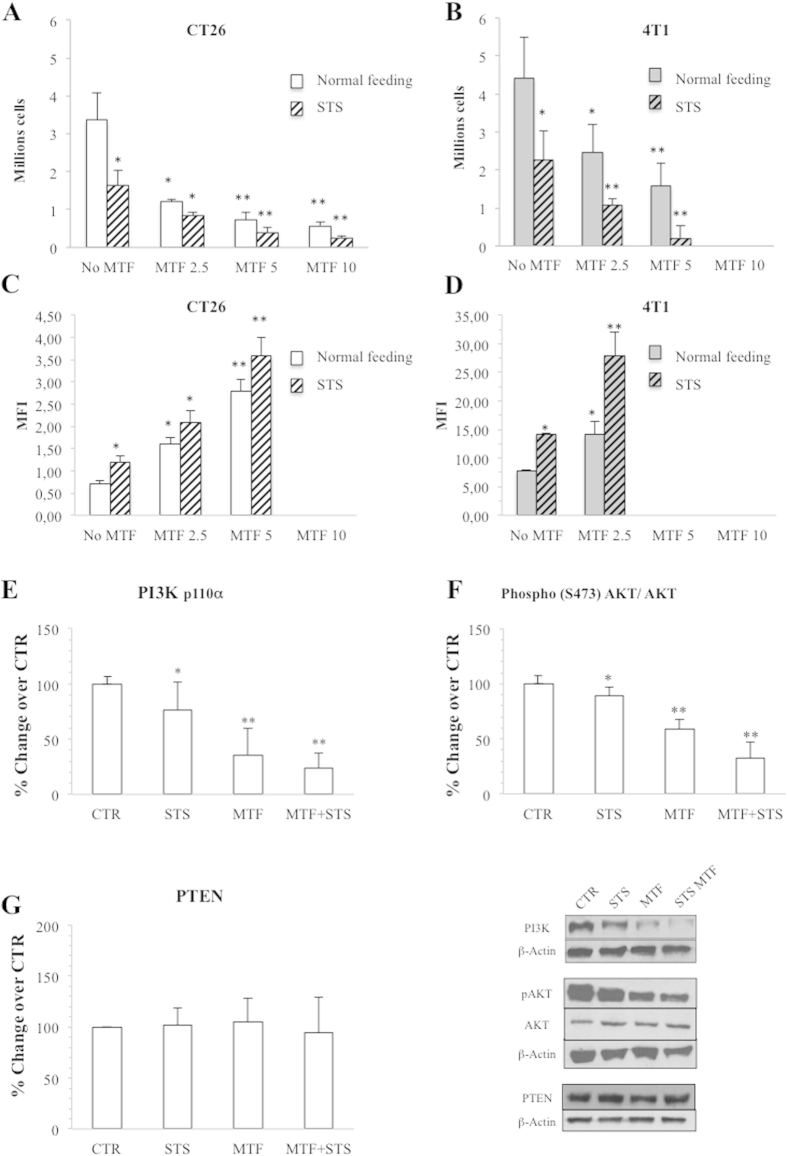
Cancer biological response to energy depletion. (**A**) In CT26, cell viability progressively decreased with increasing MTF doses. This response was further amplified by STS (dashed columns). (**B**) This effect was more evident in 4T1 cells in which the cytotoxicity of MTF + STS was already massive at 5 mM MTF. (**C,D**) Proliferation rate was reduced under each condition with a largely additive effect of MTF and STS, both in CT26 (**C**) and 4T1 (**D**) cells. At Western blot evaluation in CT26 cells, STS, MTF and mostly merged treatment caused a decrease in PI3K (**E**) and pAKT expression (**F**) despite a stable expression of PTEN in all conditions (**G**). Data are presented as mean ± SD; (*p < 0.05; **p < 0.01 statistical differences vs controls).

Since glucose consumption is critical for cancer cell survival and growth, we verified whether the profound impairment in cell energy asset and glycolytic rate did eventually hamper cell viability and proliferation. Actually, the effect of MTF and STS was confirmed by this analysis: MTF caused a cytotoxic effect that was amplified by STS in CT26 (Fig. 7A) and mostly in 4T1 cells, in which massive cell death occurred under combined treatment already at 5 mM MTF (Fig. 7B). Synergism analysis did not identify a statistically significant interaction. Finally, the reduction in cell viability was paralleled by a decrease in proliferation rate in both models of colon (Fig. 7C) and breast (Fig. 7D) carcinoma.

This biological response closely agreed with a down-regulation of pathways promoting cancer growth that were tested in CT26 cells. In fact, MTF markedly reduced PI3K expression (Fig. 7E) and AKT phosphorylation (Fig. 7F) with a further, though relatively modest, enhancement induced by STS (Fig. 7E,F) despite stable levels of phosphatase and tensin homolog (PTEN) under all study conditions (Fig. 7G). Again, synergism analysis did not identify a statistically significant interaction.

## Discussion

The present study confirms the hypothesis that combining MTF and STS profoundly depletes cancer energy asset interfering with different steps of the same metabolic pathways: glycolysis and OXPHOS. This impairment was particularly evident in cancer cells in which merged treatment caused an evident cytotoxic effect as well as a marked decrease of *in vitro* cell proliferation rate and *in vivo* tumour growth.

The interaction between the two interventions on FDG uptake resulted from the combined interference on different enzymes regulating glycolytic pathway: MTF hampered HK II function while STS affected catalytic activity of PFK and PK. A similar consideration applied to OXPHOS that offered different targets to each treatment: MTF inhibited respiratory activity directly hampering Complex I function, while STS uncoupled electron transport chain and ATP synthase increasing ROS generation. As a result, cancer cells exposed to merge treatment coupled massive energy depletion with a significant oxidative damage. The obvious consequence of this impairment was a significant cytotoxic effect associated with a reduction in proliferating rate in both colon and breast carcinoma.

In agreement with previous experience^18^,^19^, FDG uptake showed a dose-dependent decrease under MTF. This response was more evident in 4T1 than in CT26 cells and was further amplified by STS in both cell lines. The paradoxical reduction in glucose uptake occurred despite ATP depletion and was independent from variations in GLUT1 abundance. On the contrary, it was at least partially explained by an impaired glucose phosphorylation caused by MTF interference on HK II catalytic pocket^18^. The relevance of this effect was confirmed by both relative stability of enzyme availability and decrease in intra-cellular G6P concentration.

Differently from biguanide, STS down-regulated glucose consumption selectively interfering with the glycolysis rate limiting step PFK and the downstream reaction catalysed by PK. This concept was confirmed by the observation that STS-induced block in downstream reactions prevented the MTF-induced decrease in G6P level and abolished the marked increase in lactate release caused by MTF alone. Besides this direct interference, both treatments impaired HK II activity with a further mechanism. The catalytic function of this enzyme is largely empowered by its p-AKT-dependent link with outer mitochondrial membrane^20^. In agreement with response of p-AKT, immunofluorescence analysis documented that both MTF and STS, and even more their association, prevented this mitochondrial binding and thus the direct enzyme access to mitochondrial ATP for glucose phosphorylation. Accordingly, HK II activity impairment was relatively underestimated by our enzymatic assays and largely contributed to the reduction in glycolytic flux^21^.

The most recognized cellular effect of MTF is related to its capability to hamper mitochondrial Complex I activity^12^,^22^. This drug action was largely confirmed in our study and implied a virtual OCR abolition through Complex I-III-IV pathway in both cancer cell lines. As an obvious consequence, MTF determined a severe reduction in cell energy asset and ATP synthesis. The decreased OXPHOS rate was coherent with the observed increase in glycolytic end product (lactate), whose extracellular release represents a basic physiological response to restore NADH oxidation to NAD^+^ as to maintain glycolytic flux^23^. By contrast, STS prevented this biguanide effect and decreased lactate release regardless MTF presence. This response was only partially explained by a concomitant down-regulation in LDH activity while it was coherent with the accelerated OCR induced by STS in both cell lines. On the other hand, the mismatch between increased OXPHOS rate and reduced ATP content was explained by the uncoupling of electron transport chain and ATP synthase function resulting in increased ROS generation and consequent mitochondrial membrane damage^24^,^25^. Interestingly, this response persisted virtually unmodified under MTF, confirming the contribution of Complex II to the oxidative damage^26^, besides the well-described role of Complexes I and III^27^,^28^.

The severe energy depletion associated with an accelerated ROS generation under merged treatment eventually resulted in a profound reduction in cancer cell proliferation and viability that closely agreed with the downregulation of PI3K-Akt pathway^29^. More importantly, the relevance of this direct metabolic effect was confirmed *in vivo* in which the virtual flattening of tumour growth curve was at least partially enhanced by the whole body adaptation to the metabolic action of both stressors. In agreement with previous experience, neither abolition of food intake during STS nor MTF treatment alone significantly affected serum glucose level that, instead, was significantly reduced only in mice exposed to merged treatment. This observation closely fits with the well-documented anti-diabetic action of biguanide that limits gluconeogenesis^9^,^30^ and impairs G6Pase activity in liver and kidney cells as to increase their glycogen content^31^ while decreasing glucose delivery into the bloodstream.

Actually, cancer metabolic response to the merged treatment tended to decrease over time and was less evident at PET#2 than one week after tumour implantation. However, this finding was largely apparent due to the continuous nature of MTF administration as opposed to the transient application of STS, suggesting that the biguanide actually prevented the rebound phase of glucose consumption during re-feeding period. On the other hand, the marked reduction in lesion growth observed under merged treatment with respect to MTF alone, suggests the presence of further factors amplifying the anticancer potential besides the interference on glucose availability.

In this line, the possible therapeutic potential of combining MTF and STS was corroborated by several observations: on one hand, the selectivity of STS and MTF action on cancer cells was indicated by the opposite response of normal fibroblasts in which all interventions increased FDG uptake. On the other hand, the combined treatment virtually abolished tumour growth and profoundly lessened total cancer glucose consumption *in vivo* being well tolerated in the whole group of 18 treated mice. Obviously, the high MTF dose needed and STS are difficult to extend to the clinical setting. However, the virtual abolition of cancer growth observed in mice supports the potential of targeting energy metabolism in cancer treatment. In this line, these data point out the need to identify specific ways to carry the drug directly into the lesion or molecules effective at lower concentration to improve therapy effectiveness.

## Methods

### Chemicals

MTF was provided by Sigma-Aldrich (St. Louis, MO, USA). FDG was produced according to standard methodology. Daily quality controls always documented adequate standards and, in particular, a radiochemical purity ≥98%^32^.

### Animal Models

All animal experiments were reviewed and approved by the Licensing and Ethical Committee of our Institute and by the Italian Ministry of Health. six- weeks-old female BALB/c mice purchased from Charles River Laboratories (Lecco,,Italy), housed under specific pathogen-free conditions. The caloric content of the normal chow was distributed as 58% carbohydrate, 12% fat, and 30% protein, and normal food consumption was maintained during the whole experiments for all groups except for starved mice that interrupted the normal diet twice before each PET studies for 48 hours. All animals were allowed free access to water. Metformin was orally administered by diluting the drug in autoclaved drinking water at a concentration of 3 mg/mL according to a procedure approximately accounting for a dose of 750 mg/Kg/die^19^,^33^,^34^.

As a preliminary study, 2 × 10^5^ CT26 colon cancer cells were inoculated subcutaneously in the dorsal hip of 12 syngeneic mice that were followed without any other manoeuvre for four weeks under control conditions or under merged treatment. For the imaging study, inoculum was performed with the same number of either CT26 or 4T1 cells.

Each model was subsequently divided into four groups of six animals: “control” group did not receive any treatment and were kept under standard conditions for the whole duration of the study; “STS” group were submitted to 48 hours STS (absence of food) before each imaging; “MTF” group received metformin treatment for the whole duration of the study and “MTF + STS” group received the combined treatment. Cancer volume was determined by using external caliper and tumor volume was calculated using the following equation: tumor volume (mm3) = (length × width × height) × π/6, expressing length, width and height in mm. Mice were euthanized by CO2 asphyxiation at the end of the study.

### Experimental micro-PET scanning protocol

*In vivo* imaging was performed according to a protocol validated in our lab^19^,^34^. Mice were weighted and anesthesia was induced by intra-peritoneal administration of ketamine/xylazine (100 and 10 mg/kg, respectively). Serum glucose level was tested and animals were positioned on the bed of a dedicated micro-PET system (Albira, Carestream Inc, US) whose two-ring configuration permits to cover the whole animal body in a single bed position. A dose of 3–4 MBq of FDG was then injected through a tail vein, soon after start of a list mode acquisition lasting 50 minutes.

### Image processing

Acquisition was reconstructed using the following framing rate: 10 × 15 secs, 5 −× 30 secs, 2 × 150 secs, 6 × 300 secs, 1 × 600 secs). PET data were reconstructed using a maximal likelihood expectation maximization method (MLEM). An experienced observer, unaware of the experimental type of analyzed mouse, identified a volume of interest (VOI) in the left ventricular chamber. Then, the computer was asked to plot the time-concentration curve within this VOI throughout the whole acquisition to define tracer input function. Whole body FDG clearance (in ml × min-1) was calculated using the conventional stochastic approach as the ratio between injected dose and integral of input function from 0 to infinity, fitting the last 20 minutes with a mono-exponential function^35^. Further VOIs were drawn over cancer lesions to measure maximal standardized uptake value (SUV), i.e. the most commonly accepted index of tissue FDG uptake, expressed as the fraction of injected tracer dose normalized for body weight.

Cancer and normal tissues glucose consumption (metabolic rate of glucose - MRGlu) was expressed in nM X min-1 X g-1 and was estimated in these last VOIs according to Gjedde-Patlak^36^ graphical analysis by using the routine of a dedicated software (PMOD, Zurich, Switzerland). Briefly, the software utilizes the input function to transform the original tissue activity measurements by fitting the data in each voxel with the slope of the regression line defined by the model. In all cases, lumped constant value was set at 1.

### Cell lines and culture conditions

CT26, 4T1 and human fibroblasts, were purchased from ATCC (LGC Standards S.r.l., Milan, Italy) and cultured in DMEM medium (Invitrogen, Monza, Italy) containing 2 g/l glucose and supplemented with 1% L-glutamine, penicillin/streptomycin, nonessential amino acids and 10% fetal bovine serum (FBS) (all from Sigma Aldrich, Milan, Italy). All treatments were performed at 37 °C under 5% CO_2_. Short-term starvation (STS) consists in glucose and in serum restriction obtained by culturing the cells in DMEM medium containing 0.5 g/l glucose and 1% FBS for 48 hours. MTF incubation was performed with different drug concentrations (2,5; 5 and 10 mM) for 24 and 48 hours, in combination or not with STS.

### FDG Uptake Evaluathion

Labeling was performed incubating 10^6^ cells with FDG according to a procedure validated in our laboratory^18^. Immediately before the experiment, glucose free medium was added with two mL PBS containing FDG at a concentration of 37 KBq/mL. Tracer exposure was maintained for 60 minutes at 37 °C. Thereafter, uptake process was stopped by adding 4 ml of PBS before centrifugation at 450 g for 10 min. Supernatant was removed and cell pellet re-suspended in 1 ml of saline buffer. Free and bound activities were thus simultaneously counted using a Packard Cobra II gamma counter (Packard, Meriden, CT) with a 10% energy window centered at 511 KeV. FDG retention was measured as the ratio between bound and total radioactivity. In all cases, labeling procedure did not affect cell viability as documented by trypan blue staining.

### Proteomic analysis

Cellular peptides were prepared as described in details in supplementary section. The samples in the different experimental conditions were analyzed by liquid chromatografy-tandem mass spectrometry (LC-MS/MS). Raw mass spectrometric data were analyzed with the MaxQuant software (version 1.4.1.2)^37^.

### Western blotting

Western blot experiments were performed accordingly to the standard procedure^38^. We tested the following antibodies: anti-GLUT1, anti-HKII, anti-PI3K (Cell Signaling), anti-PTEN (Millipore), anti-Phospho-AKT (Ser473) and anti-β-actin (Cell Signaling).

### Spectrophotometric enzymes assay

Hexokinase (HK), Phosphofructokinase (PFK), glucose 6 phosphate (G6P), pyruvate kinase (PK) and lactic dehydrogenase (LDH) were assayed spectrophotometrically in a double beam spectrophotometer (UNICAM UV2, Analytical S.n.c., Italy) as previously described^18^. The activity assay of the four redox Complexes in the CT26 and 4T1 cells were measured on 50 μg of total protein as described in the supplementary methods paragraph.

### Co-localization experiments

Intracellular localization of HK was studied on cells cultured on glass coverslips and treated with MitoTracker probe (Life Technologies Ltd, Monza MB, Italy), rabbit anti-HKII (C64G5) (primary antibodies (Euroclone) and then with a goat anti-Rabbit Alexa Fluor 488 secondary antibody (Molecular Probes Eugene, OR, USA). Results were analyzed using an Olympus (Olympus Optical) laser-scanning microscope FV500 equipped with an Olympus IX81 inverted microscope and Argon ion 488 nm, He-Ne 543 nm, and He-Ne 633 nm lasers. Digital images were acquired through a PLAPO 60 × objective, with the Fluoview 4.3b software program. Images were acquired sequentially as single transcellular optical sections. Spatial co-localization was analyzed by Image J 1.34f software (NIH).

### Evaluation of ATP concentration

ATP concentration was measured in a luminometer (Lumi-Scint, Bioscan) by the luciferin/luciferase chemiluminescent method. The reactions employed are described in supplementary methods.

### Oxygraphic measurements, and ROS production

O2 consumption was measured using a thermostatically controlled oxygraph apparatus equipped with amperometric electrode (Microrespiration, Unisense A/S, Århus, Denmark) as described in supplementary section.

ROS production was evaluated by 2′, 7′–dichlorofluorescein diacetate (DCFDA, Invitrogen) staining and acquisition/ analysis in a Gallios cytometer (Becton Dickinson).

### Cell viability and proliferation

Cell viability was evaluated by Trypan blue (Sigma Aldrich) exclusion test. To asses proliferation, cancer cells were labeled with 20 μM CarboxyfluoresceinSuccinimidyl ester (CFSE) (InVitrogen) following the manufacture’s instructions. Samples were acquired in a Gallios cytometer and analysed using Kaluza software.

### Statistical analysis

The data are presented as mean ± standard deviation (SD). For comparison between different groups, the Null hypothesis was tested by analysis of variance (ANOVA) for multiple groups. Synergism was tested using factorial experiment design in which the interaction factor was tested using the univariate analysis of the general linear regression model as described by Slinker^39^. Statistical significance was considered for p values p < 0.05. All analyses were performed using SPSS software package, 20.0.0 release (IBM, Armonk, NY)

## Additional Information

**How to cite this article**: Marini, C. *et al.* Divergent targets of glycolysis and oxidative phosphorylation result in additive effects of metformin and starvation in colon and breast cancer. *Sci. Rep.* **6**, 19569; doi: 10.1038/srep19569 (2016).

## Supplementary Material

Supplementary Information

## References

[b1] Vander Heiden M., Cantley L. C. & Thompson C. B. Understanding the Warburg effect: the metabolic requirements of cell proliferation. Science 324, 1029–1033 (2009). 19460998 10.1126/science.1160809 PMC2849637

[b2] Macheda M. L., Rogers S. & Best J. D. Molecular and cellular regulation of glucose transporter (GLUT) proteins in cancer. J. Cell Physiol. 202, 654–662 (2005). 15389572 10.1002/jcp.20166

[b3] Vleugel M. M. *et al.* Differential prognostic impact of hypoxia induced and diffuse HIF-1alpha expression in invasive breast cancer. J. Clin. Pathol. 58, 172–177 (2005). 15677538 10.1136/jcp.2004.019885 PMC1770566

[b4] Lee C. *et al.* Fasting cycles retard growth of tumors and sensitize a range of cancer cell types to chemotherapy. Sci. Transl. Med. 4, 124–127 (2012). 10.1126/scitranslmed.3003293 PMC3608686 22323820

[b5] Hanahan D. & Weinberg R. A. Hallmarks of cancer: the next generation. Cell 144, 646–74 (2011). 21376230 10.1016/j.cell.2011.02.013

[b6] Trachootham D., Alexandre J. & Huang P. Targeting cancer cells by ROS-mediated mechanisms: a radical therapeutic approach ? Nat. Rev. Drug. Discov. 8, 579–591 (2009). 19478820 10.1038/nrd2803

[b7] Diehn M. *et al.* Association of reactive oxygen species levels and radioresistance in cancer stem cells. Nature 458, 780–783 (2009). 19194462 10.1038/nature07733 PMC2778612

[b8] Raffaghello L. *et al.* Starvation-dependent differential stress resistance protects normal but not cancer cells against high-dose chemotherapy. Proc. Natl. Acad. Sci. USA 105, 8215–8220 (2008). 18378900 10.1073/pnas.0708100105 PMC2448817

[b9] Madiraju A. K. *et al.* Metformin suppresses gluconeogenesis by inhibiting mitochondrial glycerophosphate dehydrogenase. Nature 510, 542–546 (2014). 24847880 10.1038/nature13270 PMC4074244

[b10] Zhou G. *et al.* Role of AMP-activated protein kinase in mechanism of metformin action. J. Clin. Invest. 108, 1167–1174 (2001). 11602624 10.1172/JCI13505 PMC209533

[b11] Shaw R. J. *et al.* The kinase LKB1 mediates glucose homeostasis in liver and therapeutic effects of metformin. Science 310, 1642–1646 (2005). 16308421 10.1126/science.1120781 PMC3074427

[b12] Owen M. R., Doran E. & Halestrap A. P. Evidence that metformin exerts its anti-diabetic effects through inhibition of Complex 1 of the mitochondrial respiratory chain. Biochem. J. 348, 607–614 (2000). 10839993 PMC1221104

[b13] Viollet B. *et al.* Cellular and molecular mechanisms of metformin: an overview. Clin. Sci. 122, 253–270 (2012). 22117616 10.1042/CS20110386 PMC3398862

[b14] Birsoy K., Sabatini D. M. & Possemato R. Untuning the tumor metabolic machine: targeting cancer metabolism: a bedside lesson. Nat. Med. 18, 1022–1023 (2012). 22772555 10.1038/nm.2870

[b15] Zordoky B. N., Bark D., Soltys C. L., Sung M. M. & Dyck J. R. The anti-proliferative effect of metformin in triple-negative MDA-MB-231 breast cancer cells is highly dependent on glucose concentration: implications for cancer therapy and prevention. Biochim. Biophys. Acta 1840, 1943–1957 (2014). 24462945 10.1016/j.bbagen.2014.01.023

[b16] Hadad S. M., Hardie D. G., Appleyard V. & Thompson A. M. Effects of metformin on breast cancer cell proliferation, the AMPK pathway and the cell cycle. Clin. Transl. Oncol. 16, 746–752 (2014). 24338509 10.1007/s12094-013-1144-8

[b17] El-Mir M. Y. *et al.* Dimethylbiguanide inhibits cell respiration via an indirect effect targeted on the respiratory chain Complex I. J. Biol. Chem. 275, 223–228 (2000). 10617608 10.1074/jbc.275.1.223

[b18] Salani B. *et al.* Metformin impairs glucose consumption and survival in Calu-1 cells by direct inhibition of hexokinase-II. Sci. Rep. 3, 2070 (2013). 23797762 10.1038/srep02070 PMC3691576

[b19] Marini C. *et al.* Direct inhibition of hexokinase activity by metformin at least partially impairs glucose metabolism and tumor growth in experimental breast cancer. Cell Cycle 12, 3490–3499 (2013). 24240433 10.4161/cc.26461 PMC3906335

[b20] Robey R. B. & Hay N. Mitochondrial hexokinases, novel mediators of the antiapoptotic effects of growth factors and Akt. Oncogene 25, 4683–4696 (2006). 16892082 10.1038/sj.onc.1209595

[b21] Vander Heiden M. G. *et al.* Growth factors can influence cell growth and survival through effects on glucose metabolism. Mol. Cell. Biol. 21, 5899–5912 (2001). 11486029 10.1128/MCB.21.17.5899-5912.2001 PMC87309

[b22] Mithieux G., Guignot L., Bordet J. C. & Wiernsperger N. Intrahepatic mechanisms underlying the effect of metformin in decreasing basal glucose production in rats fed a high-fat diet. Diabetes 51, 139–143 (2002). 11756333 10.2337/diabetes.51.1.139

[b23] Rovetto M. J., Whitmer J. T. & Neely J. R. Comparison of the effects of anoxia and whole heart ischemia on carbohydrate utilization in isolated working rat hearts. Circ. Res. 32, 699–711 (1973). 4715192 10.1161/01.res.32.6.699

[b24] Lambert A. J. & Brand M. D. Reactive oxygen species production by mitochondria. Methods. Mol. Biol. 554, 165–181 (2009). 19513674 10.1007/978-1-59745-521-3_11

[b25] Turrens J. F. Mitochondrial formation of reactive oxygen species. J. Physiol. 552, 335–344 (2003). 14561818 10.1113/jphysiol.2003.049478 PMC2343396

[b26] Paranagama M. P. *et al.* Contribution of the FAD and quinone binding sites to the production of reactive oxygen species from Ascaris suum mitochondrial Complex II. Mitochondrion 10, 158–165 (2010). 20006739 10.1016/j.mito.2009.12.145

[b27] Loschen G., Flohé L. & Chance B. Respiratory chain linked H(2)O(2) production in pigeon heart mitochondria. FEBS Lett. 18, 261–264 (1971). 11946135 10.1016/0014-5793(71)80459-3

[b28] Takeshige K. & Minakami S. NADH- and NADPH-dependent formation of superoxide anions by bovine heart submitochondrial particles and NADH-ubiquinone reductase preparation. Biochem. J. 180, 129–135 (1979). 39543 10.1042/bj1800129 PMC1161027

[b29] Cairns R. A., Harris I. S. & Mak T. W. Regulation of cancer cell metabolism. Nat. Rev. Cancer 11, 85–95 (2011). 21258394 10.1038/nrc2981

[b30] Meyer F., Ipaktchi M. & Clauser. H. Specific inhibition of gluconeogenesis by biguanides. Nature 213, 203–204 (1967). 6030591 10.1038/213203a0

[b31] Ota S. *et al.* Metformin suppresses glucose-6-phosphatase expression by a Complex I inhibition and AMPK activation-independent mechanism. Biochem. Biophys. Res. Commun. 388, 311–316 (2009). 19664596 10.1016/j.bbrc.2009.07.164

[b32] European Pharmacopoeia 5th edition, Directorate for the Quality of Medicines (EDQM), Council of Europe, Strasbourg Cedex, France, Supplement 5.1, 5.2 (2005).

[b33] Phoenix K. N., Vumbaca F. & Claffey K. P. Therapeutic metformin/AMPK activation promotes the angiogenic phenotype in the ERalpha negative MDA-MB-435 breast cancer model. Breast Cancer Res. Treat. 113, 101–111 (2009). 18256928 10.1007/s10549-008-9916-5 PMC2606917

[b34] Massollo M. *et al.* Metformin temporal and localized effects on gut glucose metabolism assessed using 18F-FDG PET in mice. J. Nucl. Med. 54, 259–266 (2013). 23287574 10.2967/jnumed.112.106666

[b35] Iozzo P. *et al.* 18F-FDG assessment of glucose disposal and production rates during fasting and insulin stimulation: a validation study. J. Nucl. Med. 47, 1016–1022 (2006). 16741312

[b36] Patlak C. S., Blasberg R. G. & Fenstermacher J. D. Graphical evaluation of blood-to-brain transfer constants from multiple-time uptake data. J. Cereb. Blood Flow Metab. 3, 1–7 (1983). 6822610 10.1038/jcbfm.1983.1

[b37] Cox J. & Mann M. MaxQuant enables high peptide identification rates, individualized p.p.b.-range mass accuracies and proteome-wide protein quantification. Nat. Biotechnol. 26, 1367–1372 (2008). 19029910 10.1038/nbt.1511

[b38] Marimpietri D. *et al.* Combined therapeutic effects of vinblastine and rapamycin on human neuroblastoma growth, apoptosis, and angiogenesis. Clin. Cancer Res. 13, 3977–3988 (2007). 17606732 10.1158/1078-0432.CCR-06-2757

[b39] Slinker B. K. The statistics of synergism. J. Mol. Cell. Cardiol. 30, 723–731 (1998). 9602421 10.1006/jmcc.1998.0655

