# Supplementary material for: Divergent targets of glycolysis and oxidative phosphorylation result in additive effects of metformin and starvation in colon and breast cancer
Source: Sci Rep. 2016 Jan 22;6:19569. doi: 10.1038/srep19569 (PMC4726140; doi:10.1038/srep19569)
Supplement: Supplementary Information [file srep19569-s1.pdf]

Divergent targets of glycolysis and oxidative phosphorylation result in additive effects of metformin and starvation in colon and breast cancer

*Cecilia Marini[1], *Giovanna Bianchi[2], Ambra Buschiazzo[3], Silvia Ravera[4]; Roberto Martella[2]; Gianluca Bottoni[3]; Andrea Petretto[5]; Laura Emionite[6]; Elena Monteverde[3]; Selene Capitanio[3], Elvira Inglese[5]; Marina Fabbi[7]; Francesca Bongioanni[3]; Lucia Garaboldi[3]; Paolo Bruzzi[8], Anna Maria Orengo[3]; Lizzia Raffaghello[2]; Gianmario Sambuceti[3].

[1] CNR Institute of Molecular Bioimaging and Physiology (IBFM), Milan, Section of Genoa, Genoa, Italy
[2]Oncology lab, Istituto Giannina Gaslini, Genoa, Italy
[3]Nuclear Medicine, Department of Health Sciences, University of Genoa and IRCCS AOU San Martino-IST, Genoa, Italy
[4]Biochemistry Lab, Department of Pharmacy, University of Genoa, Genoa, Italy
[5]Core facility, Istituto Giannina Gaslini, Genoa, Italy
[6]Animal facility, IRCCS AOU San Martino-IST, Genoa, Italy
[7]Integrated oncological therapies, IRCCS AOU San Martino-IST, Genoa, Italy
[8]Statistics and Epidemiology Unit, IRCCS AOU San Martino-IST, Genoa, Italy

Keywords: cancer metabolism, metformin, starvation, glycolysis, oxidative phosphorylation.

The authors have no potential conflicts of interest to disclose.
* Both CM and GB acted as first author.

Address for correspondence:
Cecilia Marini, MD, PhD,
C/o Nuclear Medicine
IRCCS AOU San Martino-IST,
160143- Genoa,
Italy
Email: Cecilia.Marini@unige.it

Suppl. Table 1: Body weight in grams and serum glucose level in each study group at the two different scan times

		Weight (grams)		Serum glucose (mmol/L)	
		**PET 1**	**PET 2**	**PET 1**	**PET 2**
**CTR**	CT26 (n=7)	18,60 ± 1,48	17,71 ± 1,23	4,26 ± 1,22	3,50 ± 2,59
	4T1 (n=7)	16,17 ± 0,75	17,55 ± 0,56	4,71 ± 0,74	4,74 ± 0,88
**STS**	CT26 (n=7)	16,20 ± 0,47	15,40 ± 1,94	3,58 ± 0,87	3,13 ± 1,00
	4T1 (n=7)	13,45 ± 0,27	14,34 ± 0,58	4,19 ± 1,18	4,80 ± 2,55
**MTF**	CT26 (n=7)	15,91 ± 0,61	17,31 ± 1,05	4,24 ± 1,42	3,50 ± 1,21
	4T1 (n=7)	16,36 ± 0,78	17,66 ± 0,78	4,40 ± 1,20	5,14 ± 1,48
**MTF + STS**	CT26 (n=7)	13,08 ± 0.76*	14,75 ± 0.94*	2,64 ± 1.00*	2,96 ± 0,16
	4T1 (n=7)	13,74 ± 0.36*	13,45 ± 0.59*	3,84 ± 0.71*	3,40 ± 0.85*

*=p<0.05 vs corresponding control value

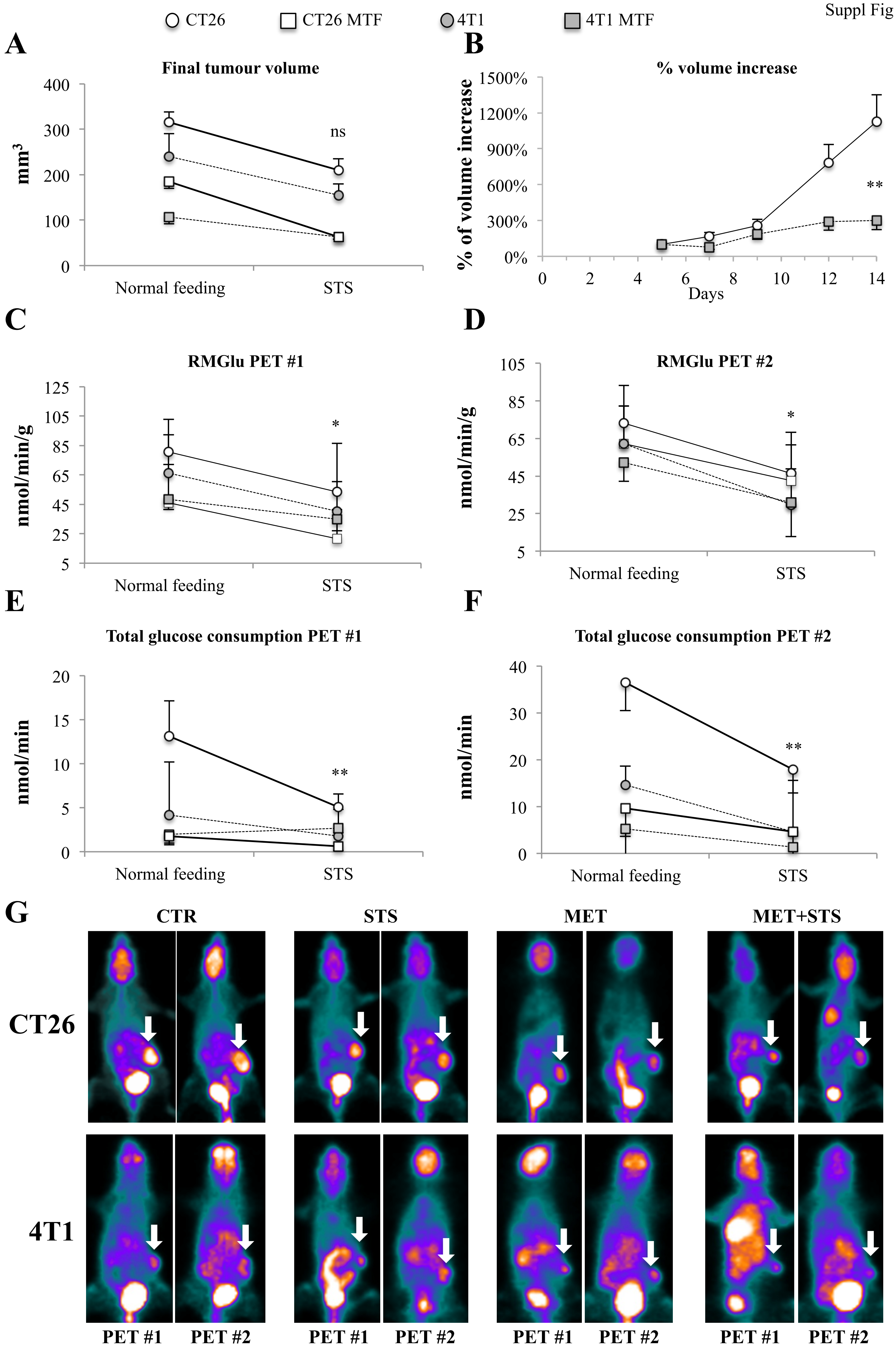
CT26
CT26 MTF
4T1
4T1 MTF
A
Final tumour volume
mm³
400
300
200
100
0
ns
Normal feeding
STS
B
% volume increase
% of volume increase
1500%
1200%
900%
600%
300%
0%
**
0
2
4
6
8
10
12
14
Days
C
RMGlu PET #1
nmol/min/g
125
105
85
65
45
25
5
*
Normal feeding
STS
D
RMGlu PET #2
nmol/min/g
105
85
65
45
25
5
*
Normal feeding
STS
E
Total glucose consumption PET #1
nmol/min
20
15
10
5
0
**
Normal feeding
STS
F
Total glucose consumption PET #2
nmol/min
40
30
20
10
0
**
Normal feeding
STS
G
CTR
STS
MET
MET+STS
CT26
4T1
PET #1
PET #2
PET #1
PET #2
PET #1
PET #2
PET #1
PET #2

Suppl. Figure #2

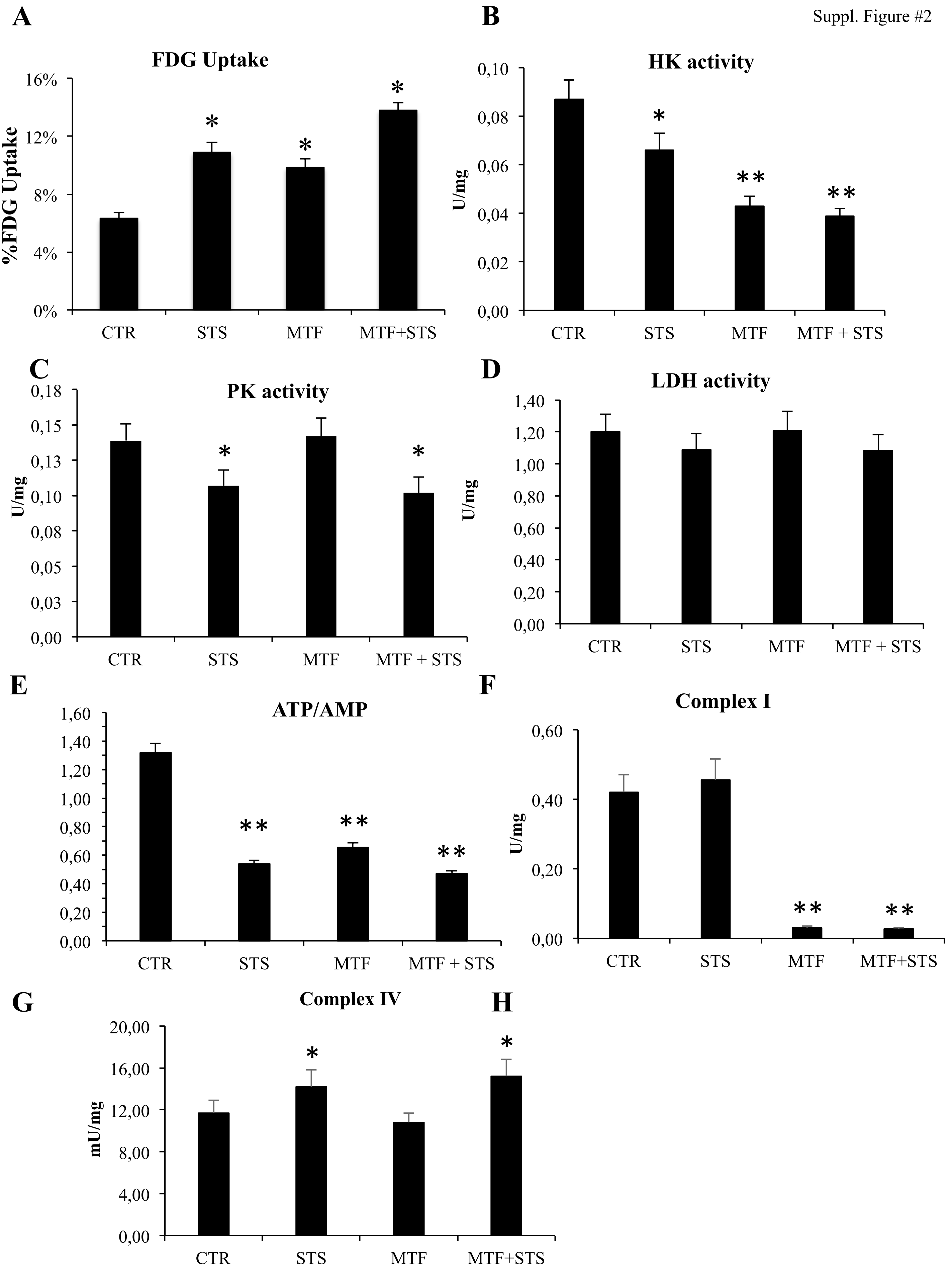

Suppl Fig #3

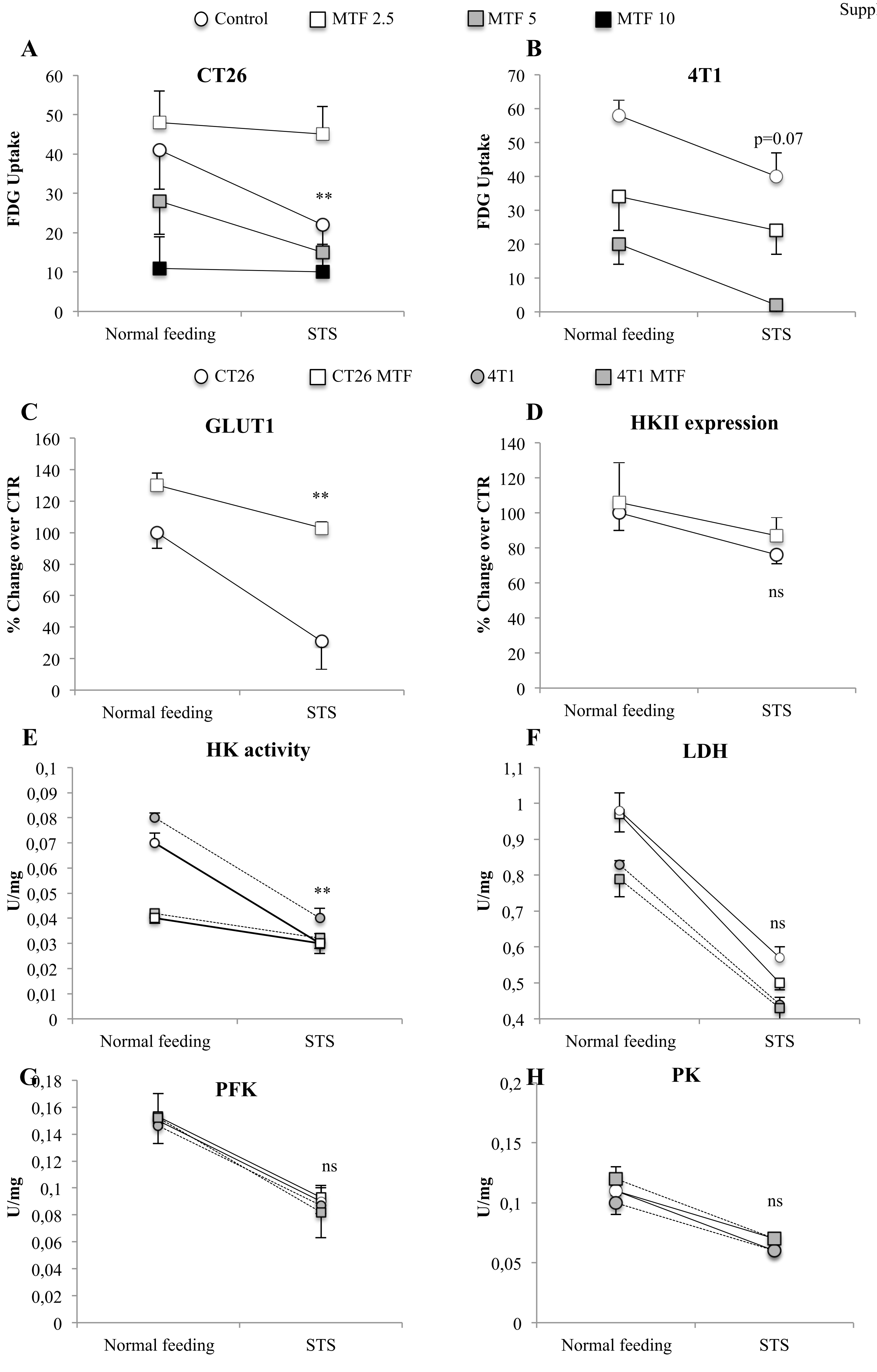

Control
MTF 2.5
MTF 5
MTF 10
A
CT26
FDG Uptake
60
50
40
30
20
10
0
**
Normal feeding
STS
B
4T1
FDG Uptake
70
60
50
40
30
20
10
0
p=0.07
Normal feeding
STS
CT26
CT26 MTF
4T1
4T1 MTF
C
GLUT1
% Change over CTR
160
140
120
100
80
60
40
20
0
**
Normal feeding
STS
D
HKII expression
% Change over CTR
140
120
100
80
60
40
20
0
ns
Normal feeding
STS
E
HK activity
U/mg
0,1
0,09
0,08
0,07
0,06
0,05
0,04
0,03
0,02
0,01
0
**
Normal feeding
STS
F
LDH
U/mg
1,1
1
0,9
0,8
0,7
0,6
0,5
0,4
ns
Normal feeding
STS
G
PFK
U/mg
0,18
0,16
0,14
0,12
0,1
0,08
0,06
0,04
0,02
0
ns
Normal feeding
STS
H
PK
U/mg
0,2
0,15
0,1
0,05
0
ns
Normal feeding
STS

A

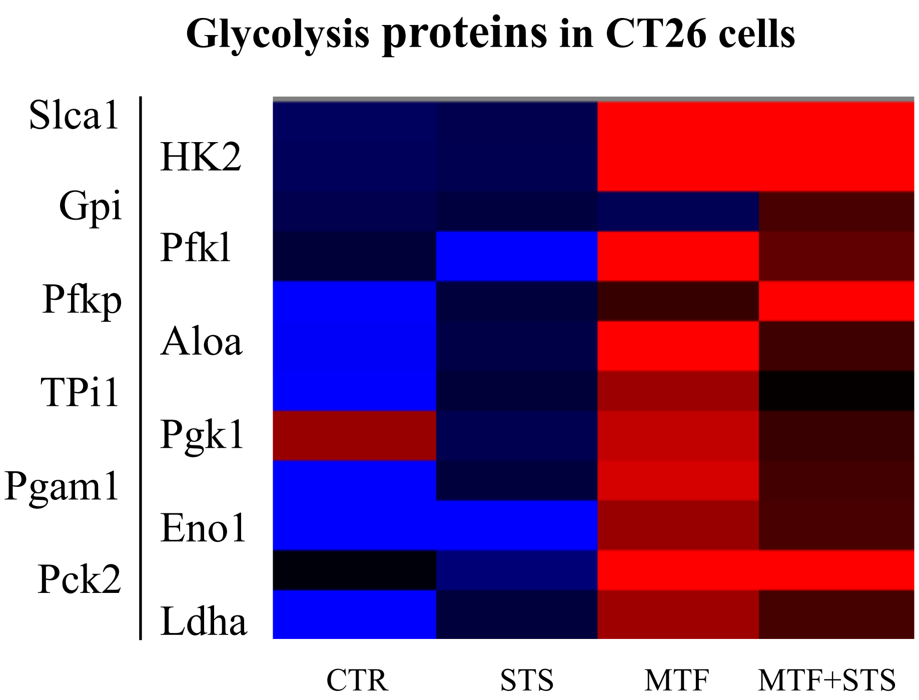
Glycolysis proteins in CT26 cells
Slca1
Gpi
Pfkp
TPi1
Pgam1
Pck2
HK2
Pfkl
Aloa
Pgk1
Eno1
Ldha
CTR
STS
MTF
MTF+STS

B

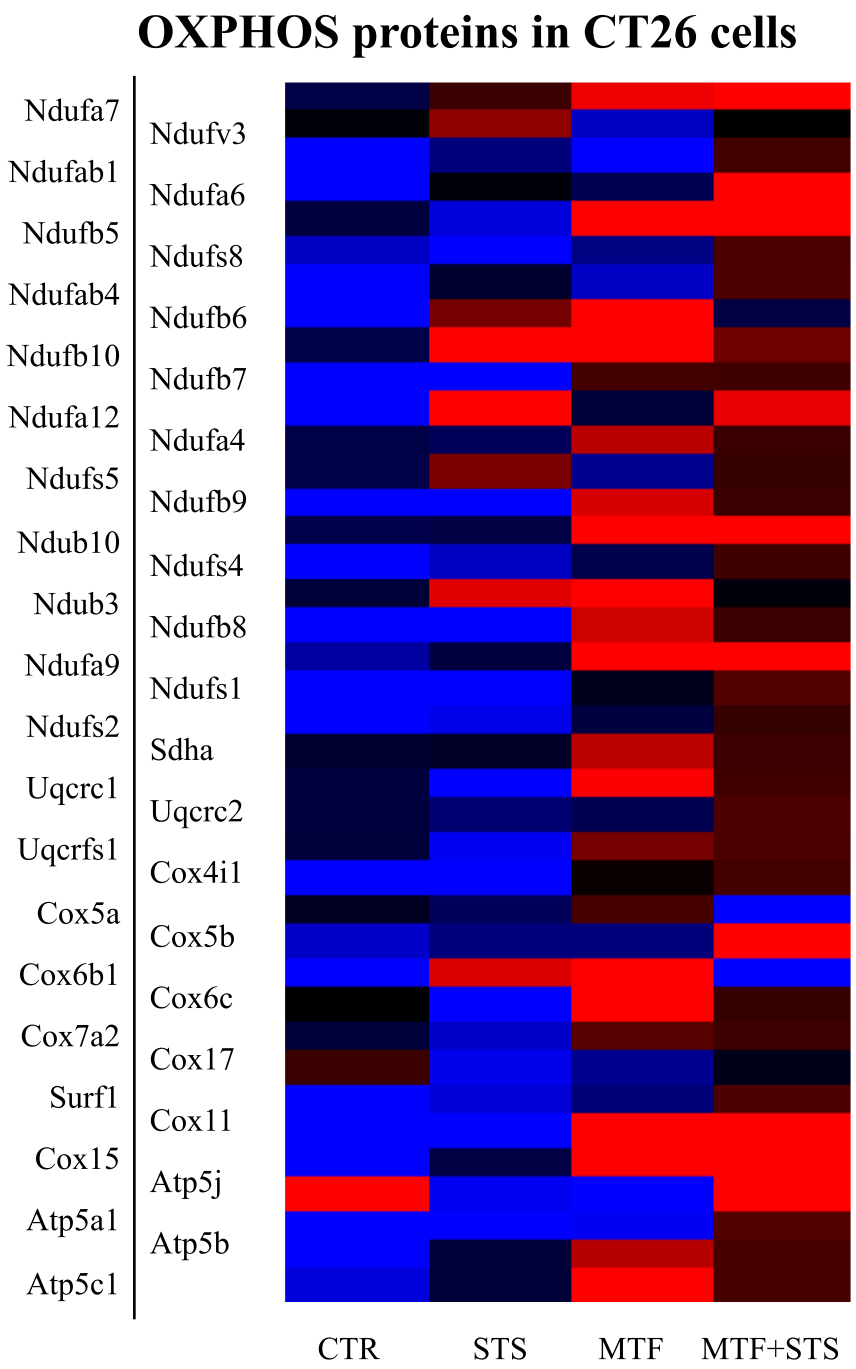
OXPHOS proteins in CT26 cells
Ndufa7
Ndufab1
Ndufb5
Ndufab4
Ndufb10
Ndufa12
Ndufs5
Ndub10
Ndub3
Ndufa9
Ndufs2
Uqcrc1
Uqcrfs1
Cox5a
Cox6b1
Cox7a2
Surf1
Cox15
Atp5a1
Atp5c1
Ndufv3
Ndufa6
Ndufs8
Ndufb6
Ndufb7
Ndufa4
Ndufb9
Ndufs4
Ndufb8
Ndufs1
Sdha
Uqcrc2
Cox4i1
Cox5b
Cox6c
Cox17
Cox11
Atp5j
Atp5b
CTR
STS
MTF
MTF+STS

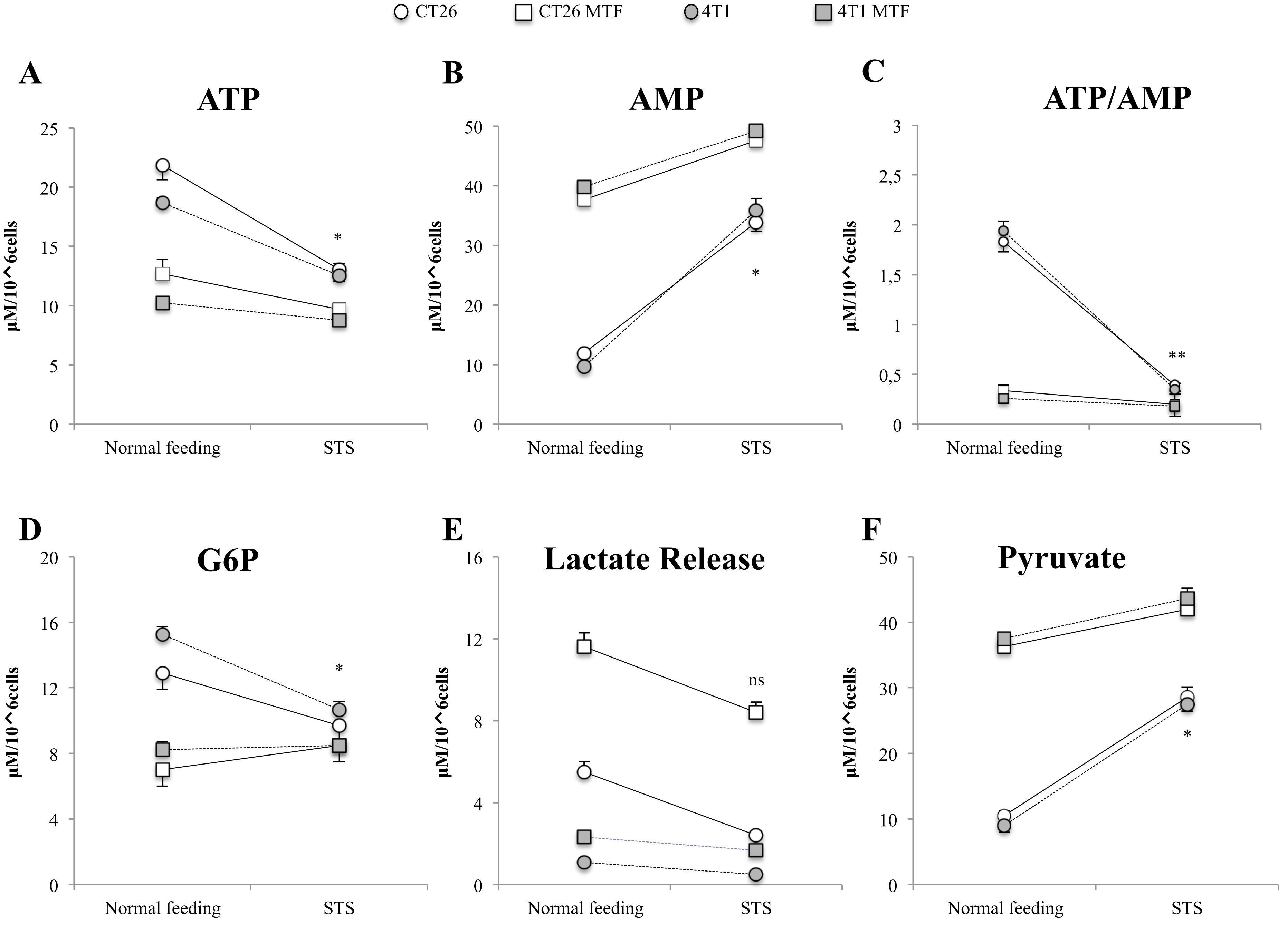

CT26
CT26 MTF
4T1
4T1 MTF
A
ATP
B
AMP
C
ATP/AMP
D
G6P
E
Lactate Release
F
Pyruvate
µM/10^6cells
Normal feeding
STS
*
**
ns

Suppl Fig #6

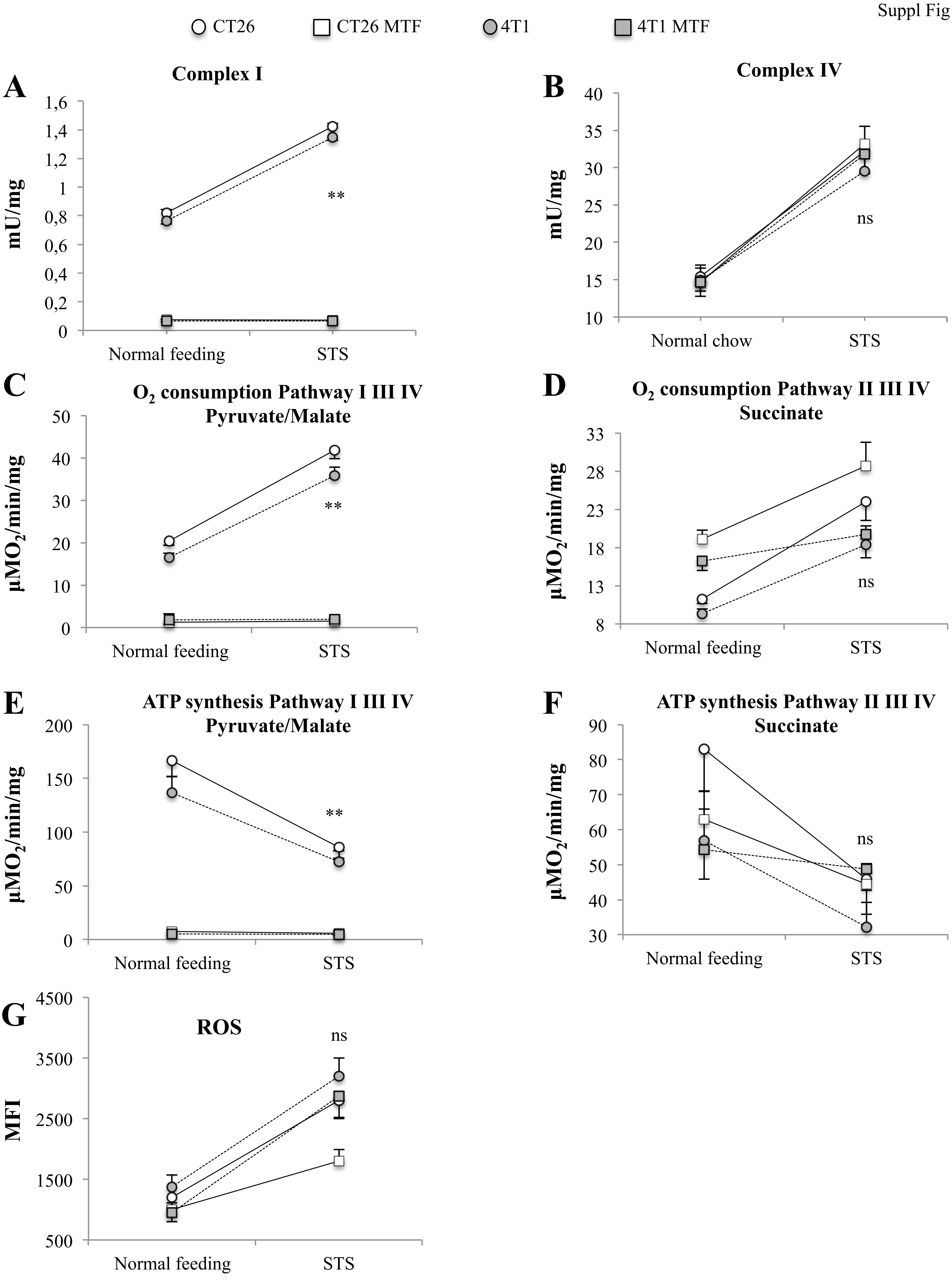
CT26
CT26 MTF
4T1
4T1 MTF
A
Complex I
mU/mg
**
Normal feeding
STS
B
Complex IV
mU/mg
ns
Normal chow
STS
C
O_2 consumption Pathway I III IV
Pyruvate/Malate
μMO_2/min/mg
**
Normal feeding
STS
D
O_2 consumption Pathway II III IV
Succinate
μMO_2/min/mg
ns
Normal feeding
STS
E
ATP synthesis Pathway I III IV
Pyruvate/Malate
μMO_2/min/mg
**
Normal feeding
STS
F
ATP synthesis Pathway II III IV
Succinate
μMO_2/min/mg
ns
Normal feeding
STS
G
ROS
MFI
ns
Normal feeding
STS

Suppl Fig #7

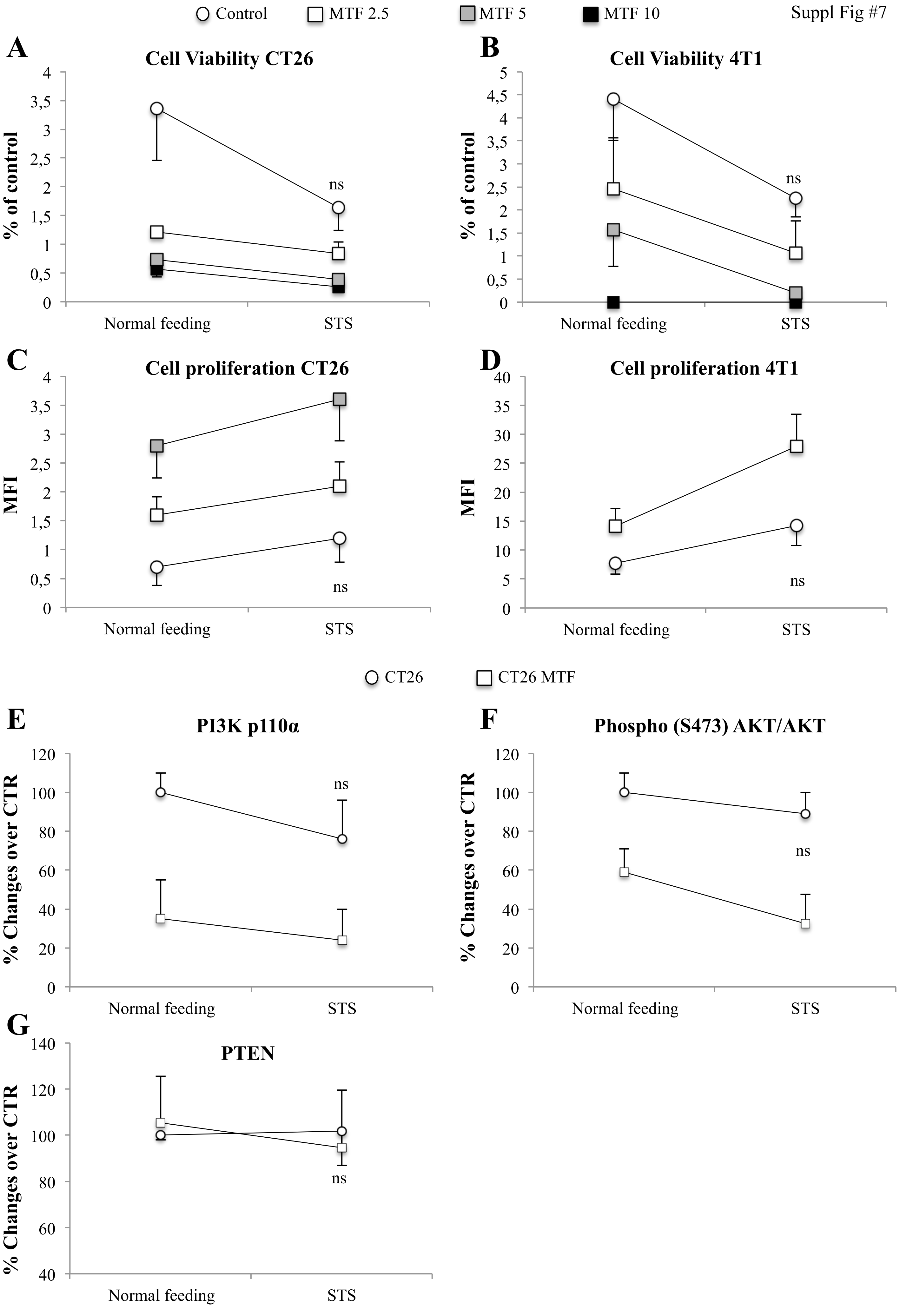
Control
MTF 2.5
MTF 5
MTF 10
A
Cell Viability CT26
% of control
0
0,5
1
1,5
2
2,5
3
3,5
4
ns
Normal feeding
STS
B
Cell Viability 4T1
% of control
0
0,5
1
1,5
2
2,5
3
3,5
4
4,5
5
ns
Normal feeding
STS
C
Cell proliferation CT26
MFI
0
0,5
1
1,5
2
2,5
3
3,5
4
ns
Normal feeding
STS
D
Cell proliferation 4T1
MFI
0
5
10
15
20
25
30
35
40
ns
Normal feeding
STS
CT26
CT26 MTF
E
PI3K p110α
% Changes over CTR
0
20
40
60
80
100
120
ns
Normal feeding
STS
F
Phospho (S473) AKT/AKT
% Changes over CTR
0
20
40
60
80
100
120
ns
Normal feeding
STS
G
PTEN
% Changes over CTR
40
60
80
100
120
140
ns
Normal feeding
STS

Supplementary methods

Proteomic analysis

Sample preparation

The cells were lysed, solubilized, denatured and reduced using Cell Extraction Buffer (Invitrogen, Cat. No. FNN0011) and containing 1% of Protease Inhibitor Cocktail (Sigma, Cat. No. P8340). The protein concentration was quantified by BCA Protein Assay Kit (Pierce, Cat.No. 23225).

The samples were processed by the FASP Protein Digestion Kit (ExpedeonInc, Cat.No. 44250). Briefly the samples were mixed with 0.3 ml of 8 M urea in 0.1 M Tris/HCl pH 8.5 (UA solution), loaded into the filtration devices, alkylated in 0.1 ml of 50 mM iodoacetamide in UA solution for 1 h in darkness at room temperature (RT). The samples were digested using sequentially 1 µg of LysC and 3 µg of Trypsin in 50 mM NaHCO3 solution at 37 °C overnight.

Peptides were collected by centrifugation of the filter units for 10 min and the filter devices were rinsed with two 40 µl washes 50 mM NaHCO3 and 50 µl 0.5 M NaCl to eliminate the hydrophobic interaction (Fraction No. 1). Furthermore in order to improve the digestion efficiency, in the same filter was added 1 µg of Glu-C in 50 mM NaHCO3 solution at 37 °C overnight and the peptides were treated as before (Fraction No. 2).

Each sample digest was desalted on StageTips and analyzed by liquid chromatography-tandem mass spectrometry (LC-MS/MS).

NanoLC setup

The peptide separation was performed with Dionex Ultimate 3000 RSLC nanoSystem.

The sample was first loaded from the sample loop onto a trapping column (2 cm × 100 µm ID, Acclaim PepMap C18, 2 µm particles, 100 Å pore size; Thermo Scientific Cat. No. 164564) using the loading solvent (98% H2O and 2% CH3CN, 0.1% formic acid) at a flow rate of 5 µl/min for 5 min. The trapping column was then switched in-line with the separation column and the peptides were eluted with increasing organic solvent at a flow rate of 300 nl/min. The peptide separation was carried out using EASY-Spray column (50 cm x 75 µm ID, PepMap C18, 2 µm particles, 100 Å pore size; Themo Scientific Cat. No. ES803), mounted on the EASY Spray Ion Source, thermostated at 40 °C. The peptides were separated and eluted with a multi-steps gradient of 5–15 % solution B (80% CH3CN and 20% H2O, 0.1% formic acid) in 10 min, 15-45% solution B in 430 min and 45-65% solution B in 15 min.

Mass spectrometer setup

The mass spectrometer LTQ-OrbitrapVelos Pro was operated in positive ionization mode.

Single MS survey scans were performed in the Orbitrap, recording a mass window between 350 and 1650 m/z using a maximal ion injection time of 250 ms. The resolution has been set to 60,000 and the automatic gain control has been set to 1,000,000 ions. The lock mass option was enabled allowing the internal recalibration of spectra recorded in the Orbitrap by polydimethylcyclosiloxane background ions (protonated (Si(CH3)2O)6); m/z 445.120025). The experiments were done in data-dependent acquisition mode with alternating MS and MS/MS experiments. The minimum MS signal for triggering MS/MS has been set to 3000 ions, with the most prominent ion signal selected for MS/MS using an isolation window of 2 Da. The m/z values of signals already selected for MS/MS were put on an exclusion list for 90 s using an exclusion window size of ±10 p.p.m. In all cases, one micro-scan was recorded. CID was done with a target value of 3,000 ions in the linear ion trap, a maximal ion injection time of 100 ms, normalized collision energy of 35%, a Q-value of 0.25 and an activation time of 10 ms. A maximum of 20 MS/MS experiments were triggered per MS scan.

Data analysis

Raw mass spectrometric data were analyzed with the MaxQuant software (version 1.4.1.2). A false discovery rate (FDR) of 0.01 for proteins and peptides and a minimum peptide length of 6 amino acids were required. A time□dependent mass recalibration algorithm was used together with the Xcalibur lock mass option for recalibration to improve the mass accuracy of precursor ions). MS/MS spectra were searched by the Andromeda search engine which is incorporated into the MaxQuant software suite against Uniprot human database (release 2013_03) combined with 248 common contaminants and concatenated with the reversed versions of all sequences. In the Fraction No. 1 for the search trypsin was chosen as enzyme specificity while in the Fraction No. 2 trypsin and Glu-C were chosen. Cysteine carbamidomethylation was selected as a fixed modification, while protein N□terminal acetylation, methionine oxidation and deamidation (N, Q), were selected as variable modifications . Maximally two missed cleavages were allowed. Initial mass deviation for the precursor ion was up to 7 ppm, and maximum allowed mass deviation for fragment ions was 0.5 Da. Protein identification required two peptides one of which had to be unique to the protein group. In order to merge the proteins, resulting from the different enzymatic digestions, the two fractions have the same name in the “Experiment Column” of the “Experimental Design Template”. Quantification in MaxQuant was performed using the built in label free quantification algorithm, enabling the ‘Match between runs’ option (time window 2 minutes).

Bioinformatic analysis

Label free experiments were analyzed with the freely available Perseus software, which includes all necessary functionalities (1). Protein groups were filtered to require in at least one experimental group three valid values. Label free intensities were logarithmized and empty values were imputed with random numbers from a normal distribution, whose

mean and standard deviation were chosen to best simulate low abundance values close to noise level. A modified Anova test with permutation based FDR statisticswas applied to filter the first results. We performed 250 permutations and required an FDR of 0.05. Hierarchical clustering of resulting proteins was performed on logarithmized intensities after z-score normalization of the data, using Euclidean distances. The Protein categorical annotation was supplied by Gene Ontology biological process, molecular function, and cellular component. All annotation was extracted from the UniProt database.

The "annotation matrix algorithm", implemented in Perseus software, and the Heatmap were used to describe the differential enrichment of specific Gene Ontology biological process terms in the samples. The software filters protein annotation terms by testing the difference among means for any protein annotation from the overall ratio distribution for all cells of the expression matrix. The statistical test is a two-dimensional non-parametric Mann–Whitney test, with a Benjamini–Hochberg multiple hypothesis testing correction that was controlled by using a false discovery rate threshold of 0.05. All categories that survived the test for at least one of the samples were converted to one row of the annotation matrix. The values for each row are the differences between groups of the Mann–Whitney test mentioned above. Fisher's exact test was performed with a false discovery rate value of 0.02.

Cytoscape (2) was used to visualize and graph protein expression data in the biological processes previously found by enrichment. Glycolisis, TCA cycle and Fatty acid metabolism were obtained from Wikipathways and KEGG integrated each other and modified based on our biological data and questions. Each enzyme was shown as rectangle in which colour match to expression value obtained from logarithmized intensities after z-score normalization of the proteomics data. The colour of enzymatic complexes is proportional to the average of the expression value of single component. The label of each protein is the correspondent gene name, while the label of the complex is the common acronym or the EC number.

Spectrophotometric enzymes assay

Complex I (NADH-ubiquinone oxidoreductase) was assayed following the reduction of ferricyanide at 420 nm. Complex II (Succinic dehydrogenase) activity was measured at 600 nm, in 2 mM EDTA, 0.2 mM ATP, 20 mM succinate, 0.5 mM cyanide, 80 μM dicloroindophenol (DCIP), 50 μM decylubiquinone, 40 μM antimycin A, 10 μM rotenone and 10 mM phosphate buffer, pH 7.2. To measure Complex III (Cytochrome c reductase) activity was followed the reduction of oxidized Cytochrome c at 550 nm Complex IV (Cytochrome c oxidase) was assayed following the oxidation of ascorbate-reduced Cytochrome c at 550 nm (3).

Bioluminescent luciferase ATP assay

ATP synthesis in CT26 cells was conducted first incubating samples for 10 min at 37 °C in 100 mM Tris/HCl (pH 7.4), 100 mM KCl, 1 mM EGTA, 2.5 mM EDTA, 5 mM $MgCl_2$, 0.2 mM di (adenosine-5') penta-phosphate, 0.6 mM ouabain, ampicillin (25 μg/ml), 5 mM KH_2PO_4 and 20 mM succinate or 0,7 mM NADH. ATP synthesis was then induced adding 0.1 mM ADP at the same pH of the mixture, to the sample. ATP concentration in each sample was measured in a luminometer (Lumi-Scint, Bioscan) by the luciferin/luciferase chemiluminescent method with ATP standard solutions between 10-9 and 10-7 M for calibration (4).

Oxygraphic measurements

An electromagnetic stirrer bar was used to mix the contents of the chamber. For each experiment 500000 cells were used. Cells were permeabilized with 0.03mg/ml digitonin for 1 min, centrifuged for 9 min at 1000 rpm and resuspended in a medium containing: 120 mM NaCl, 2 mM $MgCl_2$, 1 mM KH_2PO_4, 50 mM Tris–HCl, pH 7.4, and 25 mg/ml ampicillin. Electrode was equilibrated with the same medium before each experiment, until the oxygen consumption remained constant. Additions were conducted by Hamilton syringes, through a rubber cup in a volume of no more than 0.05 ml. 10 mM pyruvate plus 5 mM malate were added to stimulate the pathway: Complex I, III and IV. 20 mM succinate was added to stimulate the pathway: Complex II, III and IV. 0.1 mM Rotenone or 0.2 mM Antimycin A was used as inhibitors for the first and the second pathway, respectively. The respiration rate was expressed in μM O_2/min/mg (5).

References

1. http://www.perseusframework.org/Perseus_1.4.1.3.zip
2. Cline, M.S. et al. Integration of biological networks and gene expression data using Cytoscape. Nat. Protoc. **2**, 2366-2382 (2007).
3. Ravera, S. et al. Evidence for aerobic ATP synthesis in isolated myelin vesicles. Int. J. Biochem. Cell. Biol. **41**, 1581–1591 (2009).
4. Ravera, S., Panfoli, I., Aluigi, M.G., Calzia, D., Morelli, A. Characterization of Myelin Sheath F(o)F(1)-ATP synthase and its regulation by IF(1). Cell. Biochem. Biophys. **59**, 63–70 (2011).
5. Ravera, S. et al. Mitochondrial respiratory chain Complex I defects in Fanconi anemia complementation group A. Biochimie. **95**, 1828–1837 (2013).

Supplementary Figure legends:

Suppl. Figure 1: statistical analysis of synergism for the same data of Figure 1. No significant interaction could be documented for final tumour volume (panel A). Panel B displays percent increase in tumour volume in the twelve animals studied to verify toxicity profile of combined treatment. Panel C-D display MRGlu at PET #1 and #2 and the corresponding negative synergism between MTF and STS, suggesting that the two treatments act on the same metabolic pathway. Panel E-F. Similarly to panels C-D, the statistical analysis of synergism showed a negative synergism of MTF and STS on Total glucose consumption. Panel G displays images of FDG uptake conventionally as standardized uptake value (SUV). The effect of the different treatments is remarkably less evident with respect to the parametric maps obtained by Patlak approach.

Suppl Figure 2: Effect of STS and MTF on in human fibroblasts. Panel A displays that, differently from cancer cells, both 5 mM MTF and STS increased FDG uptake with this effect being even more evident under combined treatment. In panel B, HK II function was significantly reduced by all tested protocols, reaching its lowest value under MTF and merged treatment. By contrast, PK enzymatic activity was only reduced by STS regardless MTF exposure (panel C) while LDH did not show any response (panel D). The functional consequences are documented by panel E reporting the significant reduction in ATP/AMP ratio. Finally, MTF inhibition of Complex I (panel F) and STS-induced increase in complex IV function (panel G) were confirmed in these cells while. Data are presented as mean±SD. (*=$p<0.05$; **=$p<0.01$ statistical differences vs controls).

Suppl. Figure 3: Statistical analysis of synergism of STS with different MTF doses (2.5 white squares, 5 mM grey squares, 5 mM black squares). Panel A documents that MTF and STS effect on % FDG uptake was characterized by a significant synergism with a negative sign of interaction in CT26 cells. This effect was less evident in 4T1 cells, due to the higher cytotoxic effect of MTF (Panel B). A synergic effect could also be documented for GLUT1 but not for HKII expression in CT26 cells (panels C and D). By contrast, both stressors synergistically affected HK activity (again with a negative sign of interaction (panel E) but not LDH (panel F), PFK (G) and PK (H). Data are presented as mean±SD. (*=$p<0.05$; **=$p<0.01$ statistical differences vs controls).

Suppl Figure 4: Gene ontology proteomic maps in CT 26 cells. Panel A displays the effect of treatments on glycolytic enzymes; Panel B on OXPHOS components.

Suppl Figure 5: Both MTF and STS caused a significant synergistic effect with a negative sign in both CT26 and 4T1 cells, reducing ATP levels (Panel A), increasing AMP levels (Panel B) and consequently decreasing ATP/AMP ratio (Panel C). Panel D documents a significant negative interaction of both treatments in G6P levels in both cell lines. No synergism was demonstrated in lactate release (Panel E), while interaction was again negative for pyruvate levels in both CT26 and 4T1 cells (Panel F). Data are presented as mean±SD. (*=$p<0.05$; **=$p<0.01$ statistical differences vs controls).

Suppl Figure 6: Panel A documents the significant effect of MTF on Complex I inhibition without any interference by STS in both CT26 and 4T1 cells. No significant interaction was documented on Complex IV function in both cell lines (Panel B). Also OCR (pathway I-III-IV) was affected by MTF, without any interference by STS (Panel C). By contrast, STS slightly but non significantly increased OCR (pathway II-III-IV) without any interaction with MTF (Panel D). Both treatments reduced ATP synthesis (pathway I-III-IV) with a negative interaction (Panel E) without significantly altering ATP synthesis throughout pathway II-III-IV (Panel F). Finally panel G displays the absence of interaction in ROS production between the two treatments.

Suppl. Figure 7: Absence of significant interaction between STS and different MTF doses on cell viability (panels A and B) and proliferation (C and D). Panels E-F and G represent the absence of interaction in determining PI3K, phosphoAKT/AKT and PTEN levels in CT26 cells.
